# Behavioral, Biochemical, and *In Silico* Evidence for Extraction-Dependent Neuroprotective Effects of *Citrus limon* Leaf Essential Oils in Scopolamine-Challenged Zebrafish

**DOI:** 10.3390/ph19030458

**Published:** 2026-03-11

**Authors:** Salwa Bouabdallah, Ahmed Kouki, Mona H. Ibrahim, Ion Brinza, Razvan Stefan Boiangiu, Mossadok Ben-Attia, Lucian Hritcu, Amr Amin

**Affiliations:** 1 Laboratory of Environmental Biosurveillance (LR01/ES14), Faculty of Sciences of Bizerte, University of Carthage, Zarzouna 7021, Tunisia; 2 Department of Biology, Faculty of Biology, Alexandru Ioan Cuza University of Iasi, 700506 Iasi, Romania; 3 Department of Pharmaceutical Medicinal Chemistry and Drug Design, Faculty of Pharmacy (Girls), Al-Azhar University, Cairo 11754, Egypt; 4 Faculty of Sciences, “Lucian Blaga” University of Sibiu, 7–9 Ion Ratiu Street, 550024 Sibiu, Romania; 5 College of Medicine, University of Sharjah, Sharjah P.O. Box 27272, United Arab Emirates

**Keywords:** *Citrus limon*, essential oil, eco-friendly extraction, cognitive impairment, cholinergic modulation, zebrafish models, neuroinflammation

## Abstract

**Background/Objectives:** *Citrus limon* leaf essential oil (EO) is traditionally used for its calming and cognitive-enhancing properties. Although the chemical composition of *C. limon* leaf essential oils (EOs) obtained by means of hydrodistillation (HD) and solvent-free microwave extraction (SFME) has been previously characterized, the influence of the extraction method on their neuroprotective efficacy and dose–response effects remains insufficiently explored. In the present study, EOs obtained by means of HD (CEH) and SFME (CEM) were compared for their behavioral, biochemical, and *in silico* neuroprotective effects against scopolamine (SCOP)-induced cognitive and anxiety-like impairments in adult zebrafish. **Methods:** Adult Tübingen zebrafish were exposed to CEH or CEM via immersion at 10, 100, and 150 µL/L for 19 days prior to SCOP challenge (100 µM). Cognitive performance was evaluated using the Y-maze and novel object recognition (NOR) tests, while anxiety-like behavior was assessed using the novel tank test (NTT) and novel approach test (NAT). Brain acetylcholinesterase (AChE) activity and oxidative stress markers were quantified. Molecular docking analyses were conducted to investigate interactions between major EO constituents and AChE and monoamine oxidase A (MAO A). **Results:** Both CEH and CEM significantly attenuated SCOP-induced memory deficits, improved spontaneous alternation and NOR discrimination, and reduced anxiety-like behaviors. These effects were associated with AChE inhibition and restoration of redox balance. Notably, CEM generally exhibited stronger neurobehavioral and biochemical effects at comparable doses. *In silico* analyses supported these findings, revealing favorable binding affinities of key EO constituents toward cholinergic and monoaminergic targets. **Conclusions:** This study demonstrates that the extraction method influences the neuroprotective efficacy of *C. limon* leaf EOs. While both CEH and CEM exert antioxidant and cholinergic modulatory effects, CEM shows enhanced neuroprotective potential in a zebrafish model of SCOP-induced cognitive impairment, supporting the relevance of extraction-dependent biological profiling in EO-based neurotherapeutic research.

## 1. Introduction

Neurodegenerative disorders and cognitive impairments represent a growing global health burden, particularly in aging populations, with Alzheimer’s disease and related dementias being among the most prevalent conditions worldwide [1]. Increasing evidence implicates oxidative stress, neuroinflammation, and cholinergic dysfunction as key pathological mechanisms underlying cognitive decline and anxiety-related symptoms [2]. Consequently, therapeutic strategies that combine antioxidant, anti-inflammatory, and neurotransmitter-modulating properties are increasingly considered promising for the prevention or attenuation of neurobehavioral disorders.

Plant-derived essential oils (EOs) have attracted considerable attention due to their complex chemical composition and broad spectrum of biological activities, including antioxidant, anti-inflammatory, anxiolytic, and neuroprotective effects [3,4]. *Citrus* species, in particular, are rich sources of bioactive monoterpenes and oxygenated derivatives, such as limonene, linalool, geranial, and neral, which have been reported to modulate oxidative stress, cholinergic transmission, and neuronal signaling pathways [5,6].

Among them, *Citrus limon* (L.) Burm. f. leaf essential oil (EO) has been traditionally used in folk medicine for its calming properties and has shown promising biological activities in experimental models [6,7].

The biological activity of EOs is strongly influenced by their chemical composition, which in turn depends on the extraction method employed. Conventional hydrodistillation (HD) remains the reference technique recommended by international pharmacopoeias for medicinal-grade EOs. However, alternative green extraction approaches, such as solvent-free microwave extraction (SFME), have been developed to reduce extraction time, energy consumption, and thermal degradation of thermolabile compounds [7,8,9]. The chemical profiles and yields of *C. limon* leaf EOs obtained by means of HD (CEH) and SFME (CEM) have been previously characterized by our group [7]. Nevertheless, the extent to which these extraction-dependent compositional differences translate into distinct neuroprotective and behavioral outcomes has not yet been systematically investigated.

Zebrafish (*Danio rerio*) has become an established vertebrate model for investigating cognitive function, anxiety-like behavior, and neuropharmacological mechanisms, owing to its conserved neurotransmitter systems, significant genetic homology with humans, and suitability for high-throughput pharmacological screening [10].

Although scopolamine (SCOP) is clinically used as an antimuscarinic agent for gastrointestinal and motion sickness indications, its central anticholinergic effects have been widely exploited in experimental pharmacology to induce transient cognitive and anxiety-like impairments. In zebrafish, SCOP is therefore employed as a pharmacological tool to transiently disrupt cholinergic neurotransmission, enabling the evaluation of neuroprotective or cognition-modulating compounds rather than serving as a model of a specific human disease.

SCOP, a non-selective muscarinic receptor antagonist, is widely used to induce memory deficits and anxiety-like behaviors in zebrafish by disrupting cholinergic signaling and promoting oxidative stress, thereby mimicking key aspects of cognitive impairment [11,12,13]. This model provides a valuable platform for evaluating the neuroprotective potential of natural products and their underlying mechanisms of action.

While several studies have reported the anxiolytic and cognitive benefits of *Citrus*-derived EOs or their individual constituents, most investigations focus on chemical characterization or single-dose behavioral effects, without addressing how extraction-dependent variations may influence dose–response relationships, biochemical endpoints, and molecular targets. Moreover, the integration of *in vivo* behavioral assessments with biochemical markers and *in silico* approaches remains limited in the context of *Citrus* leaf EOs.

Therefore, the present study was designed to evaluate and compare the dose-dependent neuroprotective effects of *Citrus limon* leaf EOs obtained by means of CEH and CEM against SCOP-induced cognitive and anxiety-like impairments in adult zebrafish. Behavioral performance was assessed using established paradigms for memory and anxiety, while AChE activity and oxidative stress biomarkers were analyzed to elucidate underlying mechanisms. In addition, molecular docking studies were conducted to explore the interactions between major EO constituents and key neurobiological targets, namely, AChE and monoamine oxidase A (MAO A), providing mechanistic support for the observed biological effects.

## 2. Results

### 2.1. Behavioral Effects of CEH and CEM in SCOP-Treated Zebrafish

#### 2.1.1. Effects of CEH and CEM on the Y-Maze Test

**Figure 1:**
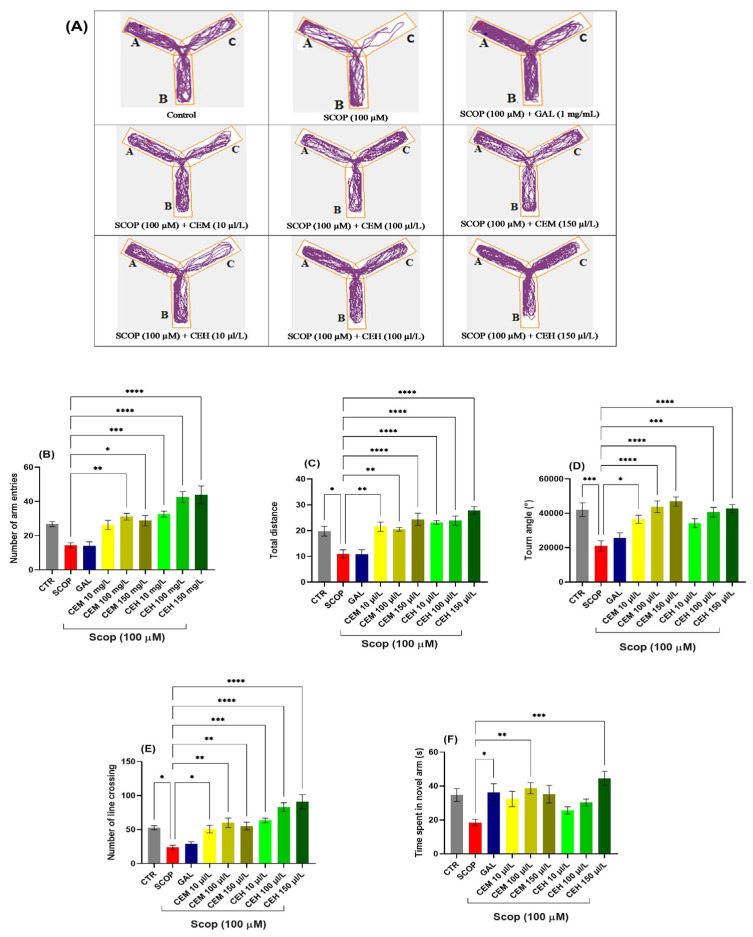
The effects of *Citrus* (CEM; CEH, 10, 100, and 150 µL/L) administration in SCOP-treated zebrafish on spatial memory and locomotor activity were assessed within the Y-maze task. (**A**) The tracking pattern of locomotion exhibited by zebrafish during the second phase of the Y-maze task was based on their respective experimental group allocations. The arms of the maze were denoted with A (start arm), B (another arm), and C (novel arm). (**B**) Number of arm entries. (**C**) Total distance (m). (**D**) Turn angle (°). (**E**) Number of line crossings. (**F**) Time spent in novel arm (S). Data are represented by means ± S.E.M. (*n* = 8). Galantamine (GAL, 1 mg/L) was used as a reference positive drug. For Tukey’s post hoc analyses * *p* < 0.05, ** *p* < 0.01, *** *p* < 0.001, and **** *p* < 0.0001. The starting point of the zebrafish’s path is denoted by the blue dot •, while the red dot • signifies the endpoint of the fish’s trajectory.

Behavioral assessment using the Y-maze test showed that SCOP (SCOP, 100 μM) significantly impaired locomotor activity and spatial memory in adult zebrafish. One-way ANOVA revealed a significant treatment effect on the number of arm entries (F (8,63) = 13.48, *p* < 0.0001) (Figure 1B). Post hoc analysis indicated that CEM at 100 µL/L significantly increased the number of arm entries (*p* < 0.01), while CEM at 150 µL/L showed a trend toward improvement that did not reach statistical significance (Figure 1B). In contrast, CEH treatment at 10, 100, and 150 µL/L produced robust increases in arm entries (*p* < 0.001, *p* < 0.0001, and *p* < 0.0001, respectively) compared to the SCOP group.

SCOP exposure also markedly reduced the total distance traveled, confirming locomotor deficits (F (8,63) = 12.31, *p* < 0.0001) (Figure 1C). Treatment with CEM (10, 100, and 150 µL/L; *p* < 0.01, *p* < 0.01, and *p* < 0.0001, respectively) and CEH (10, 100, and 150 µL/L; all *p* < 0.0001) significantly restored locomotor activity relative to the SCOP group.

Turn angle analysis further confirmed spatial memory impairment in SCOP-treated zebrafish (*p* < 0.001), which was partially reversed by galantamine (GAL; 1 mg/L; *p* < 0.05). A significant treatment effect was observed (F (8,63) = 8.64, *p* < 0.0001) (Figure 1D). CEH at 100 and 150 µL/L (*p* < 0.001 and *p* < 0.0001, respectively) and CEM at 100 and 150 µL/L (both *p* < 0.001) restored turn angle values close to control levels, whereas CEM at 10 µL/L produced no significant effect.

Line crossing behavior, expressed as the percentage of total entries, was also significantly affected by treatment (F (8,63) = 14.40, *p* < 0.0001) (Figure 1E). Both CEH and CEM at 100 and 150 µL/L significantly increased line crossings compared to SCOP-treated fish (all *p* < 0.001). Similarly, time spent in the novel arm was significantly influenced (F (8,63) = 4.05, *p* < 0.001) (Figure 1F). CEH at 150 µL/L (*p* < 0.001) and CEM at 100 µL/L (*p* < 0.01) significantly enhanced exploration of the novel arm, indicating improved short-term spatial memory.

#### 2.1.2. Novel Object Recognition (NOR) Test Effects of CEH and CEM on the Novel Object Recognition (NOR) Test

SCOP-treated zebrafish failed to discriminate between the familiar object (FO) and the novel object (NO), indicating a significant impairment of recognition memory. Tukey’s post hoc analysis confirmed a marked reduction in both preference percentage (*p* < 0.0001) and exploration time of the novel object (*p* < 0.001) compared to the control group.

**Figure 2:**
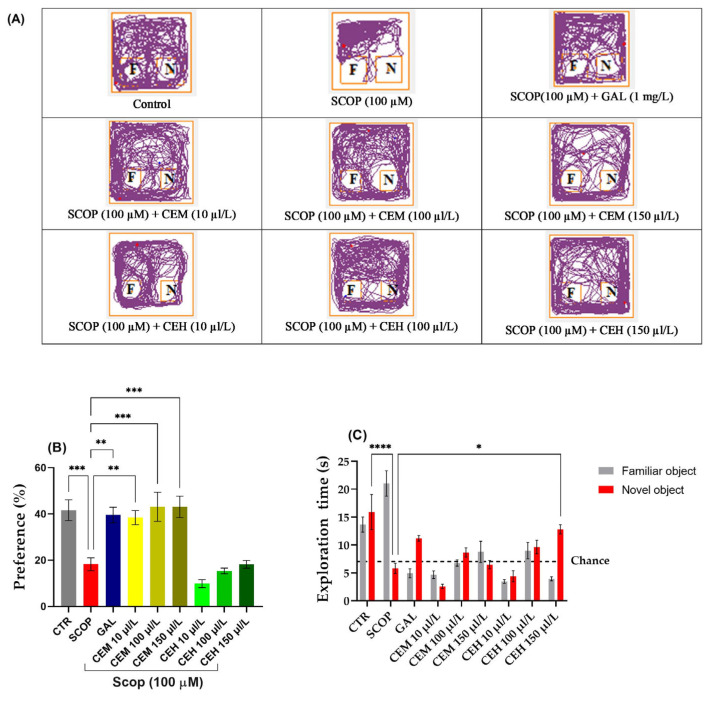
The effect of administration of *Citrus* essential oils (CEM; CEH, 10, 100, and 150 µL/L) to SCOP-treated zebrafish on recognition memory, as assessed through the object discrimination task. (**A**) The locomotor tracking patterns of the zebrafish during the object discrimination task’s testing session, according to their respective experimental groups. (**B**) The duration spent in the top (S). (**C**) The measure of preference percentage was utilized as the endpoint for evaluating recognition memory. The familiar and the novel objects are denoted as F and N, respectively. The black dashed line (chance) indicates a 50% preference. Data are represented by means ± S.E.M. (*n* = 8). Galantamine (GAL, 1 mg/L) was used as a reference positive drug. For Tukey’s post hoc analyses * *p* < 0.05, ** *p* < 0.01, *** *p* < 0.001, and **** *p* < 0.0001. The starting point of the zebrafish’s path is denoted by the blue dot •, while the red dot • signifies the endpoint of the fish’s trajectory.

Treatment with *Citrus limon* essential oils significantly restored recognition memory performance. One-way ANOVA revealed a significant treatment effect on exploration time (F (8,63) = 13.9, *p* < 0.0001) (Figure 2B). Post hoc comparisons showed that CEM significantly increased exploration of the novel object at concentrations of 10 µL/L (*p* < 0.01), 100 µL/L (*p* < 0.001), and 150 µL/L (*p* < 0.001) compared to the SCOP group.

Similarly, a significant treatment effect was observed for preference percentage (F (8,63) = 14.8, *p* < 0.0001) (Figure 2C). EO-treated zebrafish displayed preference values exceeding the chance level (50%), confirming recovery of recognition memory. No significant differences were detected between EO-treated groups and the control group, indicating a normalization of cognitive performance.

### 2.2. Effect of Citrus Essential Oils on Anxiety-like Behavior

#### 2.2.1. Novel Tank Diving Test (NTT) Effects of CEH and CEM on the Novel Tank Diving Test (NTT)

SCOP exposure significantly reduced the time spent in the top zone (*p* < 0.0001) and decreased the top-to-bottom distance ratio (*p* = 0.0001), reflecting pronounced anxiogenic behavior and reduced exploratory activity. Treatment with *Citrus limon* EOs (CEM and CEH at 10, 100, and 150 µL/L) significantly mitigated these SCOP-induced alterations.

**Figure 3:**
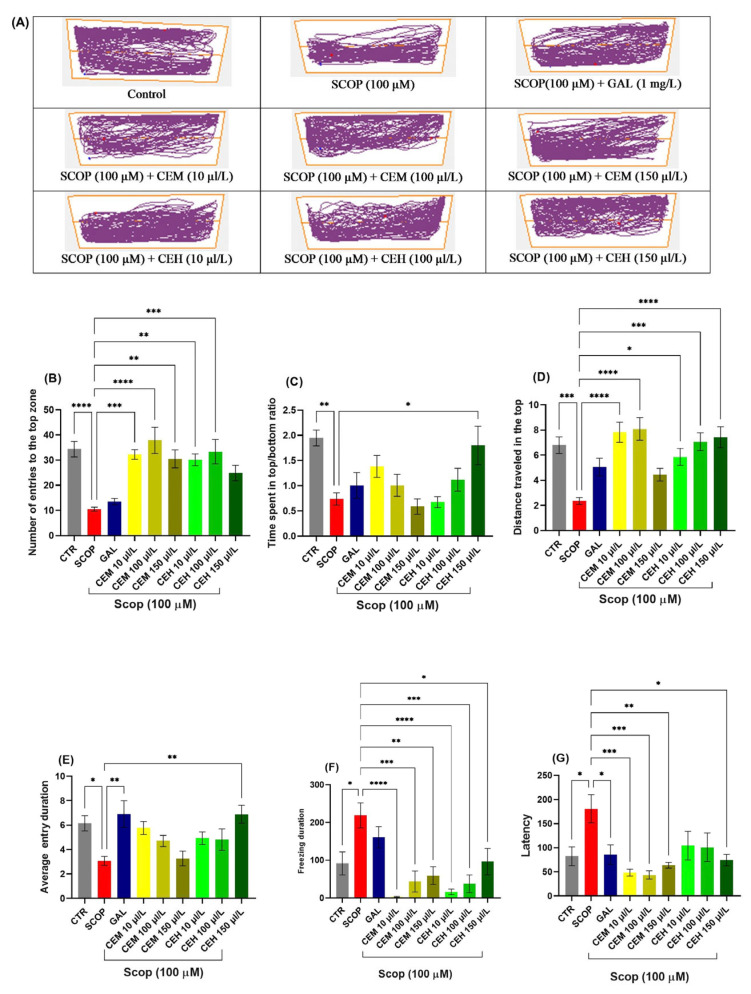
Novel tank dividing test (NTT) results for *Citrus* essential oils (CEM; CEH, 10, 100, and 150 µL/L). (**A**) Representative tracking locomotion patterns; (**B**) Number of entries to the top (s). (**C**) Time spent in the top (s); (**D**) Distance travelled in the top (m); (**E**) Average entry duration. (**F**) Freezing duration (s). (**G**) Latency. Data are expressed as means ± S.E.M. (*n* = 8). * *p* < 0.05, ** *p* < 0.01, *** *p* < 0.001, and **** *p* < 0.0001 (Tukey’s post hoc analyses). Galantamine (GAL, 1 mg/L) was used as a reference positive drug. The starting point of the zebrafish’s path is denoted by the blue dot •, while the red dot • signifies the endpoint of the fish’s trajectory.

One-way ANOVA revealed significant treatment effects on the number of entries into the top zone (F (8, 63) = 8.674, *p* < 0.0001; Figure 3B), time spent in the top zone (F (8, 63) = 13.24, *p* < 0.0001; Figure 3C), total distance traveled in the top zone (F (8, 63) = 9.56, *p* < 0.0001; Figure 3D), and average duration per top-zone entry (F (8, 63) = 6.64, *p* < 0.0001; Figure 3E). In parallel, freezing duration (F (8, 63) = 4.698, *p* = 0.0002) and latency to first entry into the top zone (F (8, 61) = 8.13, *p* < 0.0001) were significantly reduced in EO-treated groups compared to the SCOP group (Figure 3F), indicating a robust anxiolytic-like effect.

#### 2.2.2. Novel Approach Test (NAT) Effects of CEH and CEM on the Novel Approach Test (NAT)

**Figure 4:**
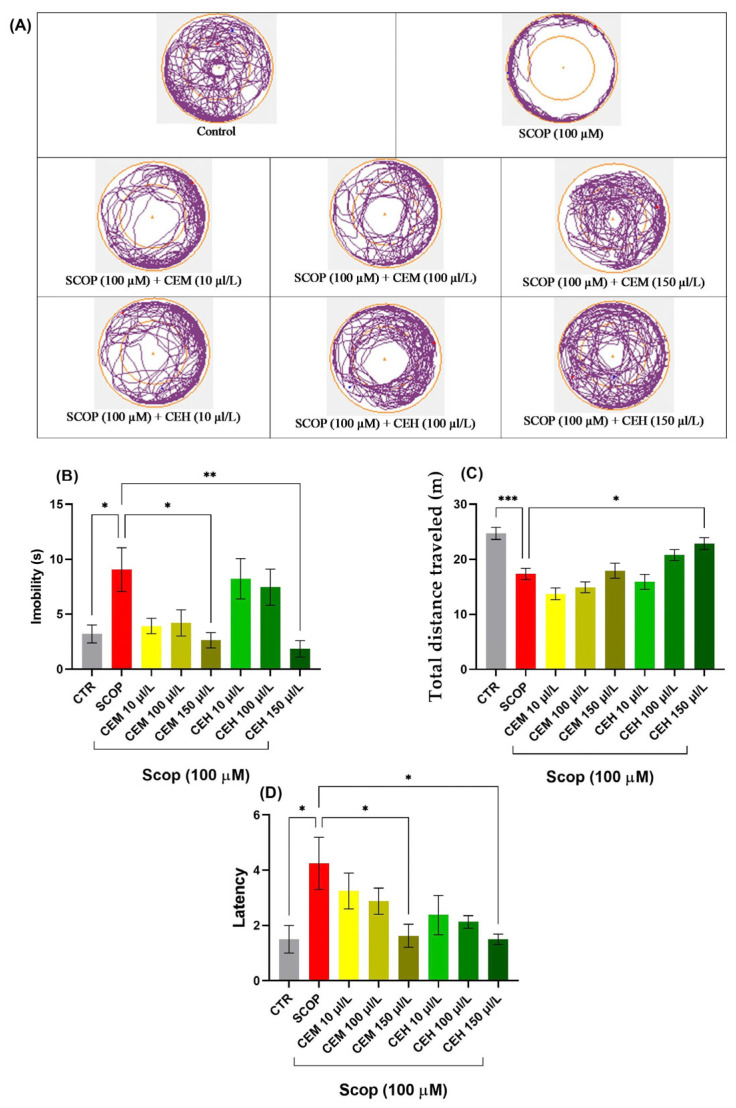
Novel approach test (NAT) results for *Citrus* essential oils (CEM; CEH, 10, 100, and 150 µL/L). (**A**) Representative tracking locomotion patterns; (**B**) Immobility (s); (**C**) Total distance travelled (m); (**D**) latency. Data are expressed as means ± S.E.M. (*n* = 8). * *p* < 0.05, ** *p* < 0.01, *** *p* < 0.001 (Tukey’s post hoc analyses). The starting point of the zebrafish’s path is denoted by the blue dot •, while the red dot • signifies the endpoint of the fish’s trajectory.

SCOP exposure induced marked locomotor and exploratory impairments, characterized by increased immobility and reduced distance traveled. One-way ANOVA revealed significant treatment effects on immobility duration (F (7, 56) = 4.458, *p* = 0.0005; Figure 4B), total distance traveled (F (7, 56) = 12.02, *p* < 0.0001; Figure 4C), and latency to approach the novel object (F (7, 56) = 2.989, *p* = 0.0079; Figure 4D).

Post hoc Tukey’s analysis showed that both CEM and CEH treatments, at all tested concentrations, significantly increased the time spent in the inner zone compared to the SCOP group (*p* < 0.0001), indicating a pronounced anxiolytic-like effect and restoration of exploratory behavior.

### 2.3. Effects on Cholinergic Function and Oxidative Stress Markers

**Figure 5:**
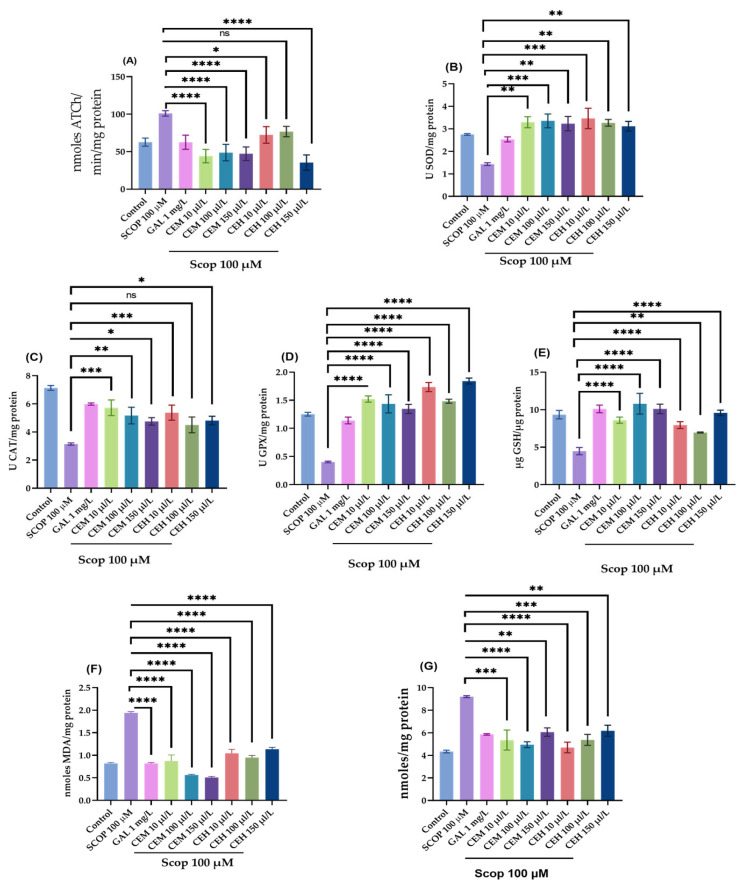
The effects of *Citrus limon* essential oils (CEM; CEH, 10, 100, and 150 µL/L) administration in scopolamine (SCOP)-treated zebrafish on (**A**) AChE, (**B**) superoxide dismutase (SOD), (**C**) catalase (CAT)-specific activities, (**D**) glutathione peroxidase (GPX), (**E**) glutathione (GSH), (**F**) malondialdehyde (MDA), and (**G**) carbonylated protein levels. Data are represented by means ± S.E.M. (*n* = 8). Galantamine (GAL, 1 mg/L) was used as a reference positive drug. For Tukey’s post hoc analyses * *p* < 0.05, ** *p* < 0.01, *** *p* < 0.001, **** *p* < 0.0001, ns = not significant.

To complement the behavioral findings, biochemical assays were conducted to assess cholinergic function and oxidative stress status in zebrafish brain tissue. One-way ANOVA revealed significant treatment effects of CEH and CEM on AChE-activity and oxidative stress markers, including AChE (F (8,18) = 16.01, *p* < 0.0001; Figure 5A), suproxyde dismutase (SOD; (F (8,18) = 6.73, *p* = 0.0004; Figure 5B), catalase (CAT; (F (8,18) = 15.12, *p* < 0.0001; Figure 5C), glutathione peroxidase (GPX; (F (8,18) = 30.81, *p* < 0.0001; Figure 5D), and reduced glutathione (GSH; (F (8,18) = 10.60, *p* < 0.0001; Figure 5E).

SCOP administration significantly reduced antioxidant enzyme activities and increased oxidative damage, as evidenced by elevated levels of malondialdehyde (MDA; F (8,18) = 49.86, *p* < 0.0001; Figure 5F) and protein carbonyls (F (8,18) = 10.00, *p* < 0.0001; Figure 5G).

CEM treatment significantly enhanced antioxidant defenses, increasing SOD activity (*p* < 0.01 at 10 and 150 µL/L; *p* < 0.001 at 100 µL/L; Figure 5B), GPX activity (*p* < 0.0001 at all tested doses; Figure 5D), CAT activity (*p* < 0.001 at 10 µL/L; *p* < 0.01 at 100 µL/L; Figure 5C), and GSH levels (*p* < 0.0001 at all doses; Figure 5E).

Similarly, CEH markedly increased GPX activity at all concentrations tested (*p* < 0.0001 at 10, 100, and 150 µL/L; Figure 5D). Both CEH and CEM significantly attenuated oxidative damage by reducing MDA levels (*p* < 0.0001 at all doses; Figure 5F) and protein carbonyl content (*p* < 0.0001 at 10 µL/L; *p* < 0.001 at 100 µL/L; *p* < 0.01 at 150 µL/L; Figure 5G).

### 2.4. Correlation Analyses Between Behavioral and Biochemical Parameters

To further elucidate the mechanistic link between behavioral outcomes and biochemical alterations, Pearson correlation analyses were performed between oxidative stress markers (CAT, SOD, GPX and MDA), AChE activity, and behavioral indices derived from the Y-maze test.

To further clarify the relationship between behavioral and biochemical outcomes, Pearson correlation analyses were conducted linking oxidative stress markers (CAT, SOD, GPX, and MDA), AChE activity, and behavioral indices from the Y-maze test.

**Figure 6:**
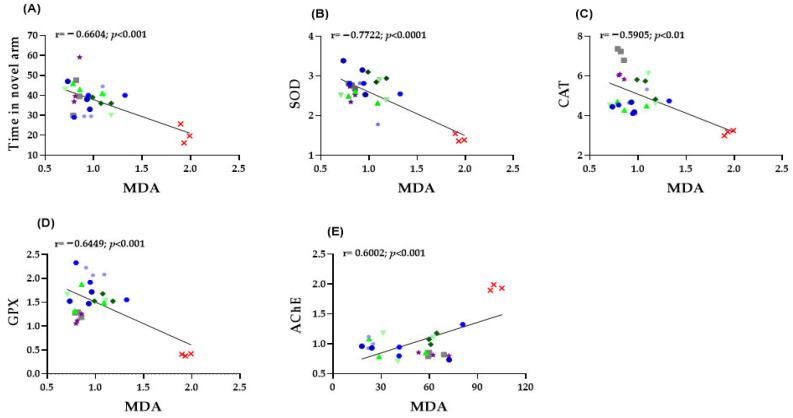
Correlation analysis between behavioral and biochemical parameters (Pearson’s correlation). Data shown are the correlation analysis between behavioral and biochemical parameters (Pearson’s correlation). (**A**) Time spent by fish in the novel arm of the Y maze vs. MDA (*n* = 8, r = −0.6604, *p* < 0.001); (**B**) SOD vs. MDA (*n*= 8, r = −0.7722, *p* < 0.0001); (**C**) CAT vs. MDA (*n* = 8, r = −0.5905, *p* < 0.01); (**D**) GPX vs. MDA (*n* = 8, r = −0.6449, *p* < 0.001); (**E**) AChE vs. MDA (*n* = 8, r = 0.6002, *p* < 0.001).

In zebrafish brain tissue, several significant correlations were identified between behavioral performance and oxidative stress markers. A moderate negative correlation was observed between MDA levels and the time spent in the novel arm of the Y-maze (r = −0.6604, *p* < 0.001; Figure 6A), indicating that increased lipid peroxidation is associated with impaired spatial memory. Moreover, MDA concentrations exhibited strong inverse relationships with the activities of antioxidant enzymes, including SOD (r = −0.7722, *p* < 0.0001; Figure 6B), CAT (r = −0.5905, *p* < 0.01; Figure 6C), and GPX (r = −0.6449, *p* < 0.001; Figure 6D.), confirming that higher oxidative stress corresponds to reduced antioxidant defense. Finally, a significant positive correlation was found between MDA levels and AChE activity (r = 0.6002, *p* < 0.001; Figure 6E), suggesting that elevated oxidative damage is closely linked to cholinergic dysfunction. Collectively, these findings highlight the strong interplay between oxidative stress, behavioral impairments, and neurochemical disturbances in the SCOP-induced zebrafish model of cognitive dysfunction.

### 2.5. Molecular Docking Analysis of Major EO Constituents

#### 2.5.1. Molecular Docking of Compounds Against Acetylcholinesterase

**Figure 7:**
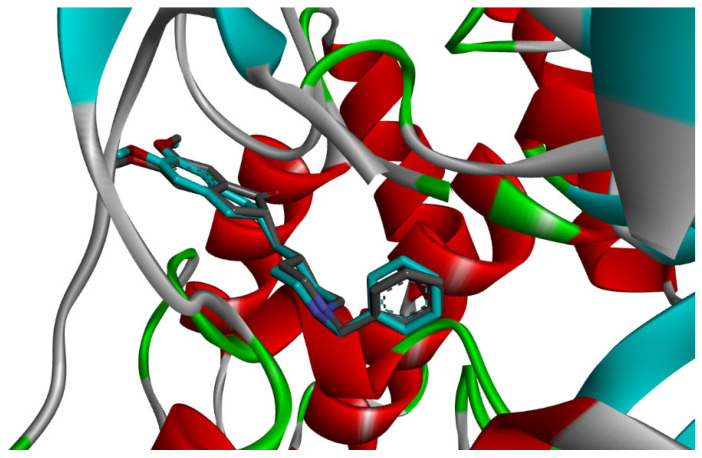
Superposition of the co-crystal ligand (Donepezil) with its docked pose in AChE (RMSD 0.43 Å).

Twenty-nine compounds were accurately identified within the active sites of the AChE enzyme, as detailed in Table S1 of the Supplementary Data. The compounds *δ*-Cadinene and (*E*)-*γ*-Bisabolene showed noteworthy interactions, as established in Table 1. The docking scores for the 29 compounds varied between −6.3 and −9.0 kcal/mol. The docking confirmation was achieved by re-docking the lead compounds into the active site. The docking process was deemed successful due to the low root-mean-square deviation (RMSD) value obtained. The root mean square deviation (RMSD) obtained for the AChE re-docking (Figure 7) was 0.43 Å, confirming the reliability of the docking protocol.

#### 2.5.2. Molecular Docking Against Monoamine Oxidase A

**Figure 8:**
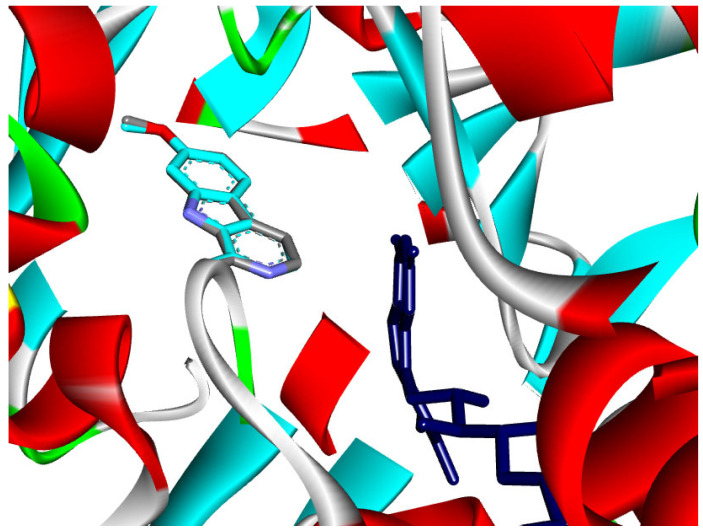
Superposition of the co-crystal ligand (Harmine) with its docked pose in the MAO A enzyme (RMSD 0.13 Å).

A total of twenty-nine various compounds were examined to determine how they influenced the Monoamine oxidase A (MAO A) enzyme (Table S2). According to the data shown in Table 2, it can be observed that the molecules (E,E)-*α*-Farnesene and (E)-*γ*-Bisabolene are involved in a considerable interaction. It was through the process of re-docking the co-crystallized ligand that we were able to confirm the docking strategy. This resulted in an RMSD of 0.13Å, which is shown in Figure 8. In total, there were 29 different compounds, and their docking scores ranged anywhere from −5.4 to −9.0 kcal/mol.

## 3. Discussion

### 3.1. Neurobehavioral Effects of Citrus limon Leaf Essential Oils in Scopolamine-Challenged Zebrafish

The present study demonstrates that *Citrus limon* leaf EOs obtained by HD and SFME significantly attenuate SCOP-induced cognitive and anxiety-like disturbances in adult zebrafish. SCOP is a well-established pharmacological agent used to induce transient cognitive impairment through disruption of central cholinergic neurotransmission, thereby reproducing key features of cholinergic dysfunction–related memory deficits commonly used in experimental models [11,12,13].

Consistent with previous reports, SCOP exposure resulted in pronounced impairments in spatial and recognition memory, accompanied by increased anxiety-like behaviors, as evidenced by altered performance in the Y-maze, NOR, NTT, and NAT tests [14,15]. These findings further validate the robustness and relevance of the SCOP-induced zebrafish model for investigating neurobehavioral dysfunction.

Treatment with both CEH and CEM markedly improved behavioral outcomes across all paradigms. Restoration of spontaneous alternation behavior, turn angle, and novel arm exploration in the Y-maze, together with normalization of object discrimination in the NOR test, confirms the ability of *C. limon* EOs to counteract SCOP-induced memory dysfunction. In parallel, the reduction of anxiety-related responses observed in the NTT and NAT tests indicates broader neuromodulatory effects extending beyond cognition.

Importantly, zebrafish treated with *C. limon* EOs exhibited behavioral performances comparable to those of control and GAL-treated groups, underscoring the pharmacological relevance of the observed effects. Collectively, these findings further support the suitability and translational value of the zebrafish model for neurobehavioral screening of natural compounds with potential therapeutic relevance [10,12,16].

### 3.2. Cholinergic and Antioxidant Mechanisms of Neuroprotection

The behavioral recovery induced by CEH and CEM was consistently accompanied by marked biochemical changes in zebrafish brain tissue, supporting a dual neuroprotective mechanism involving cholinergic regulation and redox homeostasis. SCOP is well known to induce cognitive deficits by disrupting central cholinergic neurotransmission while simultaneously promoting oxidative stress in zebrafish and other experimental models [12,13]. In the present study, SCOP markedly increased AChE activity and suppressed endogenous antioxidant defenses, including SOD, CAT, GPX, and reduced GSH, while elevating lipid and protein oxidation markers. Consistent with our findings, Liu et al. demonstrated that lemon EO significantly ameliorates cognitive dysfunction through inhibition of AChE activity and preservation of hippocampal synaptic density in an aging mouse model [17,18].

Oxidative stress is increasingly recognized as a major contributor to synaptic dysfunction, neuronal damage, and cognitive decline, particularly in Alzheimer’s disease and related neurodegenerative disorders [2,13]. Excessive production of reactive oxygen species, combined with impaired antioxidant capacity, disrupts neuronal signaling and synaptic plasticity, thereby accelerating memory impairment and behavioral alterations.

Both EO preparations significantly inhibited AChE activity, supporting the restoration of cholinergic signaling—an established therapeutic strategy for the management of cognitive disorders. This finding is consistent with structural and functional studies demonstrating the susceptibility of AChE to modulation by small bioactive molecules [14], as well as previous zebrafish investigations reporting cholinergic-targeted neuroprotection following phytochemical intervention [19,20,21]. In parallel, CEH and CEM markedly enhanced antioxidant enzyme activities and reduced oxidative damage, indicating effective attenuation of SCOP-induced oxidative stress.

Given the central role of oxidative imbalance in neurodegenerative processes, the normalization of MDA and protein carbonyl levels observed in EO-treated groups suggests preservation of neuronal integrity through maintenance of redox homeostasis. This antioxidant protection likely acts synergistically with cholinergic modulation to support synaptic function and cognitive recovery.

Beyond enzyme modulation, accumulating evidence indicates that major constituents of *Citrus limon* EOs, particularly limonene and linalool, exert synergistic antioxidant and anti-inflammatory effects by inhibiting the TLR4/NF-κB signaling pathway while activating the Nrf2/ARE axis, a master regulator of cellular redox homeostasis [22,23]. Activation of Nrf2-dependent pathways promotes the transcription of antioxidant and cytoprotective genes, preserves mitochondrial function, and limits reactive oxygen species accumulation, whereas inhibition of NF-κB signaling attenuates neuroinflammatory cascades and pro-inflammatory cytokine production.

Together, this coordinated modulation of cholinergic transmission, oxidative stress, and neuroinflammatory pathways supports neuronal survival, maintains synaptic integrity, and promotes synaptic plasticity—processes that are crucial for the recovery of SCOP-impaired cognitive functions [19,24].

### 3.3. Differential Efficacy of CEH and CEM: Extraction-Dependent Neuroprotective Outcomes

The chemical composition of *Citrus limon* leaf EOs obtained by HD (CEH) and SFME (CEM) was previously characterized using GC/MS and reported in detail by Bouabdallah et al. [7]. Accordingly, the present study focuses on the biological and *in silico* evaluation of selected major constituents rather than reiterating compositional data.

The differential neuroprotective efficacy observed between CEH and CEM can be attributed to extraction-dependent variations in their chemical profiles. Although both EOs originate from the same botanical source, SFME has been shown to favor the enrichment of oxygenated monoterpenes such as linalool, geranial, and neral, whereas HD predominantly yields oils richer in monoterpene hydrocarbons, including limonene [7].

These compositional differences are functionally relevant, as oxygenated monoterpenes are widely recognized for their enhanced antioxidant capacity, cholinergic-modulatory activity, and neuroactive potential compared to monoterpene hydrocarbons [16,17,18]. In line with this, the higher efficacy of CEM was reflected by stronger antioxidant enzyme restoration, more pronounced AChE inhibition, and superior behavioral recovery in zebrafish.

The differential neuroprotective efficacy observed between CEH and CEM can be directly attributed to extraction-dependent variations in their chemical composition, as previously characterized by GC/MS analysis [7]. Although both EOs originate from the same botanical source, SFME resulted in a higher relative abundance of oxygenated monoterpenes, including linalool, geranial, and neral, whereas HD yielded oils richer in monoterpene hydrocarbons such as limonene [7].

These compositional differences are functionally relevant, as oxygenated monoterpenes are widely recognized for their enhanced antioxidant, cholinergic-modulatory, and neuroactive properties compared to monoterpene hydrocarbons. Accordingly, previously reported GC/MS profiles [7] provide a chemical basis for the stronger antioxidant enzyme restoration, more pronounced AChE inhibition, and improved behavioral recovery observed in CEM-treated zebrafish. Overall, these findings highlight that extraction-dependent chemical fingerprints critically influence the biological efficacy of *Citrus limon* leaf EOs.

Although both extraction methods yielded biologically active EOs, CEM consistently exhibited superior efficacy across several behavioral and biochemical endpoints, particularly at higher doses. This differential activity is unlikely to result from differences in experimental exposure or administration, as treatment conditions were identical, and instead reflects extraction-dependent variations in chemical composition.

SFME is known to better preserve thermolabile and oxygenated monoterpenes compared to HD [7,9,20,21]. These compounds, including linalool and geranial, have been widely reported to exert stronger antioxidant, enzyme-modulating, and neuroactive properties than hydrocarbon monoterpenes [16,17,18]. In the present study, this compositional advantage translated into clear functional benefits.

At equivalent doses (100–150 µL/L), CEM induced a more pronounced restoration of antioxidant defenses, with SOD and CAT activities recovering to levels close to those of the control group, whereas CEH produced a more moderate, albeit significant, increase. This enhanced antioxidant efficacy was further supported by a marked reduction in lipid peroxidation; notably, CEM at 150 µL/L reduced MDA levels to values approaching baseline control levels, while CEH-treated groups showed only partial normalization.

Similarly, CEM produced a stronger inhibition of AChE activity than CEH, indicating a more efficient restoration of cholinergic neurotransmission. This enhanced effect is consistent with the higher abundance of oxygenated monoterpenes in CEM-derived oil and aligns with previous studies reporting the AChE-inhibitory and neuroprotective properties of monoterpene-rich *Citrus* EO constituents [13,24,25].

### 3.4. Molecular Docking Insights into Multi-Target Neuroprotective Mechanisms

To further elucidate the molecular basis underlying the neuroprotective effects observed in vivo, molecular docking analyses were performed against two key neurobiological targets implicated in cognitive and affective regulation: AChE and MAO-A [26].

#### 3.4.1. Docking Analysis with Acetylcholinesterase (AChE)

AChE is a serine hydrolase responsible for the hydrolysis of acetylcholine at synaptic clefts, and its inhibition represents a central therapeutic strategy for mitigating cholinergic dysfunction associated with cognitive impairment. The catalytic activity of AChE occurs within a deep active-site gorge comprising a catalytic triad (Ser203, Glu334, His447), an acyl-binding pocket, and a peripheral anionic site enriched in aromatic residues that guide substrate positioning [27,28].

**Figure 9:**
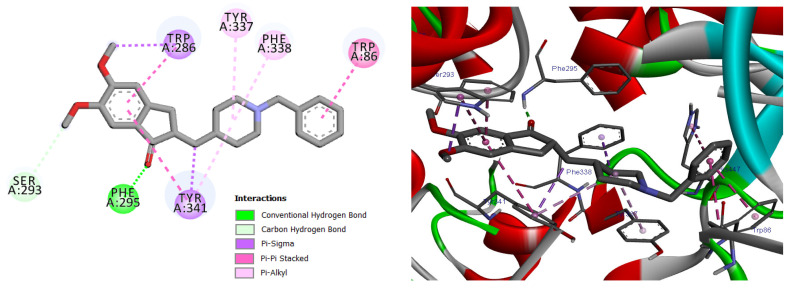
2D and 3D models of the co-crystal ligand (**Donepezil**) binding to the active site of the AChE enzyme.

Docking validation was first performed using the co-crystallized reference inhibitor donepezil, which exhibited stable binding within the AChE active-site gorge through hydrogen bonding and hydrophobic interactions with residues at both the catalytic and peripheral sites (Figure 9). This dual-site interaction pattern is consistent with the established inhibitory mechanism of donepezil and confirms the reliability of the docking protocol [27].

Subsequent docking of the 29 essential oil constituents revealed favorable binding affinities toward AChE, with predicted binding energies ranging from −6.3 to −9.0 kcal/mol (Table S1). Most compounds established stabilizing π–π stacking, π–alkyl, hydrogen bonding, and hydrophobic interactions with key residues involved in substrate recognition and catalysis, including Trp86, Tyr337, Tyr341, Trp286, Phe295, Phe297, and Ser203 [27,28].

**Figure 10:**
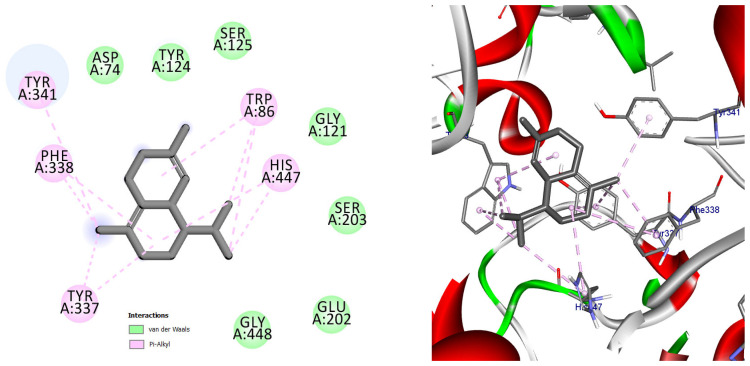
2D and 3D models of δ-Cadinene binding to the active site of the AChE enzyme.

**Figure 11:**
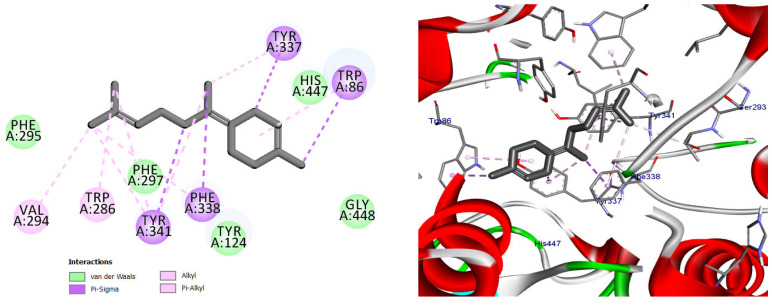
2D and 3D models of (E)-γ-Bisabolene binding to the active site of the AChE enzyme.

Among the docked compounds, δ-cadinene exhibited one of the strongest predicted affinities toward AChE and formed extensive hydrophobic and π-alkyl interactions within both the peripheral and catalytic regions of the enzyme (Figure 10). Similarly, (E)-γ-bisabolene displayed a favorable binding pose stabilized by multiple π-based and alkyl interactions with aromatic residues lining the active-site gorge, suggesting effective accommodation within the enzyme cavity (Figure 11).

Collectively, these interaction profiles support the ability of Citrus limon essential oil constituents to engage multiple functional regions of AChE, thereby contributing to the inhibition of acetylcholine breakdown observed *in vivo* [13,26].

#### 3.4.2. Docking Analysis with Monoamine Oxidase A (MAO-A)

MAO-A is a flavin-dependent enzyme involved in the oxidative deamination of monoamine neurotransmitters such as serotonin and dopamine, and its dysregulation is closely linked to anxiety- and depression-related behaviors [28,29]. The MAO-A active site comprises a hydrophobic cavity surrounding the flavin adenine dinucleotide (FAD) cofactor, which plays a central role in catalytic activity [28,29].

**Figure 12:**
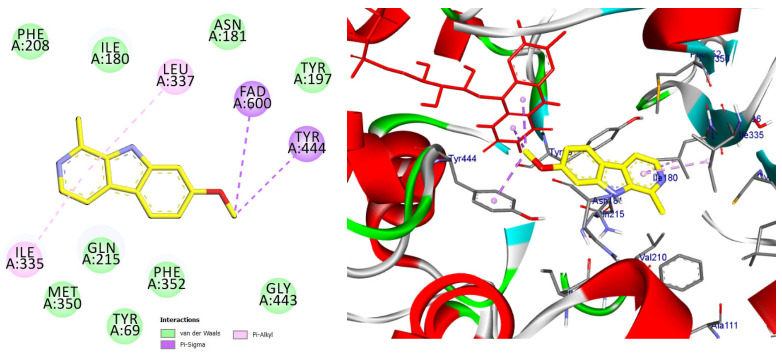
2D and 3D models of the co-crystal ligand (Harmine) binding to the active site of the MAO A enzyme.

Docking of the reference inhibitor harmine confirmed its stable positioning within the MAO-A active cavity, where it interacted with residues surrounding the FAD cofactor through π-based and hydrophobic interactions (Figure 12), consistent with its known reversible inhibitory activity.

**Figure 13:**
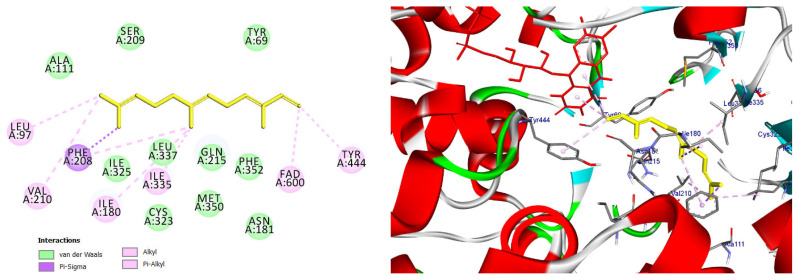
2D and 3D models of (E, E)-α-Farnensene binding to the active site of the MAO A enzyme.

**Figure 14:**
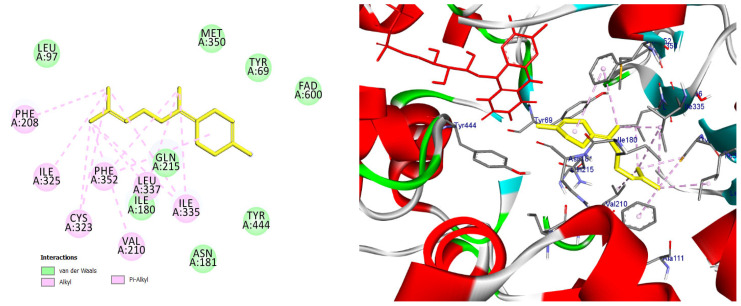
2D and 3D models of (E)-γ-Bisabolene binding to the active site of the MAO A enezym.

The majority of the EO constituents demonstrated favorable binding within the MAO-A cavity, interacting with key residues such as Tyr407, Tyr444, Ile335, Leu337, and the FAD moiety (Table S2). Among these compounds, (E,E)-α-farnesene and (E)-γ-bisabolene exhibited the strongest predicted binding affinities, exceeding that of harmine. Their docking poses revealed extensive hydrophobic stabilization within the enzyme cavity and close proximity to the FAD cofactor, suggesting potential modulation of MAO-A catalytic activity (Figure 13 and Figure 14) [21,28].

#### 3.4.3. Integration of Docking and *In Vivo* Findings

Taken together, the molecular docking results provide strong *in silico* support for the biochemical and behavioral observations obtained in vivo. The ability of *Citrus limon* EO constituents to interact with both AChE and MAO-A suggests a multi-target mechanism involving simultaneous modulation of cholinergic and monoaminergic pathways [28,29].

This dual activity likely underlies the combined cognitive-enhancing and anxiolytic effects observed in zebrafish exposed to CEH and CEM, reinforcing the neuroprotective potential of these EOs.

Predicted binding conformation of δ-cadinene within the active-site gorge of acetylcholinesterase. The compound is stabilized by multiple hydrophobic and π–alkyl interactions with functionally relevant residues at the catalytic and peripheral sites, suggesting a potential contribution to AChE inhibition.

Docking pose of (E)-γ-bisabolene in the acetylcholinesterase binding pocket. The compound establishes extensive hydrophobic and π-based interactions with aromatic residues involved in substrate recognition and stabilization, supporting its high predicted affinity toward AChE.

Binding orientation of the reference monoamine oxidase A inhibitor harmine within the MAO-A active cavity (PDB ID: 2Z5X). Harmine interacts with residues surrounding the FAD cofactor, consistent with its reversible inhibitory mechanism.

Predicted binding mode of (E, E)-α-farnesene within the MAO-A active site. The compound is positioned in proximity to the FAD cofactor and key hydrophobic residues through alkyl and π-based interactions, indicating a favorable binding profile.

Docking pose of (E)-γ-bisabolene in the MAO-A catalytic cavity. The compound forms multiple hydrophobic and π-alkyl interactions with residues critical for substrate accommodation, supporting its potential role as a MAO-A modulator.

### 3.5. Limitations and Perspectives

Despite the robust behavioral, biochemical, and *in silico* evidence supporting the neuroprotective potential of *Citrus limon* leaf EOs, several limitations of the present study should be acknowledged.

First, although the zebrafish model of SCOP-induced cognitive impairment is well validated and widely used for neurobehavioral screening, it does not fully recapitulate the chronic and multifactorial nature of neurodegenerative diseases such as Alzheimer’s disease. Consequently, extrapolation of the present findings to mammalian systems and human pathology should be made with caution.

Second, the relatively modest sample size per group, while consistent with established zebrafish behavioral studies, may limit statistical power for detecting subtle effects and dose–response relationships. Future studies incorporating larger cohorts or complementary experimental designs could further strengthen the robustness of the conclusions.

Third, the present work focused primarily on behavioral outcomes, oxidative stress markers, and enzymatic activities. While these endpoints provide valuable functional insight, additional investigations at the molecular and cellular levels—such as gene expression analysis, synaptic marker quantification, or histopathological assessment—would be necessary to confirm long-term neuroprotection and to further delineate the underlying mechanisms.

Moreover, the molecular docking analyses, although informative and mechanistically supportive, remain predictive in nature. Functional enzyme inhibition assays, pharmacokinetic studies, and blood–brain barrier permeability assessments would be required to validate the biological relevance of the predicted ligand–target interactions.

From a translational perspective, future research should aim to correlate extraction-dependent chemical composition more quantitatively with biological efficacy, potentially through fractionation approaches or targeted testing of individual oxygenated monoterpenes. Additionally, extending the investigation to mammalian models would help to clarify the therapeutic relevance of *Citrus limon* EOs and their constituents.

Overall, despite these limitations, the present study provides a comprehensive framework integrating behavioral, biochemical, and computational evidence, highlighting the importance of extraction strategy as a determinant of functional neuroprotective outcomes and paving the way for future mechanistic and translational investigations.

## 4. Materials and Methods

### 4.1. Preparation of Essential Oils

*Citrus limon* leaf EOs were extracted using two different techniques: HD and SFME. The EOs obtained by HD and SFME are hereafter referred to as CEH (*Citrus limon* essential oil from HD) and CEM (*Citrus limon* EOs from SFME), respectively. Detailed descriptions of the extraction procedures and the chemical composition of both oils have been previously reported by Bouabdallah et al. [7]. Briefly, HD involves prolonged heating of plant material in water, whereas SFME relies on microwave irradiation to rupture glandular structures, enabling a faster extraction process while limiting thermal degradation of volatile compounds.

### 4.2. Zebrafish Husbandry and Maintenance (Fish Care and Maintenance)

Wild-type zebrafish (*Danio rerio*; short-fin strain) of both sexes (50:50 ratio) were housed in the animal facility within Alexandru Ioan Cuza University of Iasi, Faculty of Biology, Romania, following standard procedures (Zebrafish Information Network) as described previously [21,30]. Briefly, zebrafish were housed in three 70-L aquariums within a recirculation system that supplied well-ventilated and dechlorinated water maintained at a controlled temperature of 26 °C ± 2. The photoperiod was set to a 14-h light to 10-h dark cycle. Water quality parameters were consistently maintained: pH at 7.5, dissolved oxygen at 7.20 mg/L, ammonium concentration below 0.004 ppm, and conductivity at 500 μS.

A total of 48 zebrafish were randomly allocated into six experimental groups (n = 8 per group) using a simple random allocation procedure. The experimental groups included a control group (system water), a scopolamine-treated group (SCOP, 100 μM), a galantamine-treated group (GAL, 1 mg/L) used as a positive control, and treatment groups exposed to *Citrus limon* EOs obtained by HD or SFME at concentrations of 10, 100, and 150 µL/L.

CEH and CEM were freshly diluted in 1% (*v*/*v*) Tween 80, selected as a vehicle based on previously published zebrafish studies employing EOs under immersion exposure in SCOP-induced models. Treatments were administered once daily by immersion for 1 h in 6 L glass tanks over 19 consecutive days.

A vehicle control group exposed to 1% (*v*/*v*) Tween 80 alone was included, and no significant behavioral or biochemical effects attributable to the vehicle were observed. Following each treatment session, fish were returned to their housing tanks containing clean system water. SCOP (100 μM) was administered 30 min prior to behavioral testing to induce cognitive impairment.

Behavioral assessments and data analysis were performed by an experimenter blinded to the treatment allocation throughout the study.

### 4.3. Behavioral Memory Assessments

#### 4.3.1. Y-Maze Test

Zebrafish response to spatial novelty was evaluated using the Y-maze task [31,32]. The spatial positioning in the maze served as an indicator of memory performance. Each fish was individually tested in a 3 L glass Y-maze tank with three arms (25 × 8 × 15 cm), randomly designated as the start arm (always open), the novel arm (opened during the test trial), and the permanently open arm. During the first training session (5 min), fish were placed in the start arm while the novel arm was closed. After 1 h, the second session (5 min) began, with the fish again starting in the start arm, but now the novel arm was open. Behavioral endpoints included total distance traveled (m), time spent in the novel arm (% of total arm time), and turn angle (°). Video recordings were analyzed using ANY-maze^®^ software v. 7.48 (Stoelting Co., Wood Dale, IL, USA).

#### 4.3.2. Novel Object Recognition (NOR) Test

On the fourth day (training phase), zebrafish were exposed to two identical objects for 10 min. One hour later (testing phase), one familiar object was replaced with a novel object for 10 min. The novel object recognition (NOR) test was conducted to assess memory-related exploratory behavior in zebrafish. Fish underwent a 5 min acclimatization period in a 30 × 30 × 30 cm tank (filled with 6 cm of water) for three consecutive days in the absence of objects, as previously described [14,15,31,32,33,34].

Memory performance was quantified as follows:

%Novel Object Exploration = time exploring NO/time exploring FO + time exploring NO ×100.

where NO refers to the novel object and FO to the familiar object. Behavioral recordings were analyzed using ANY-maze^®^ software v.7.48 (Stoelting Co., Wood Dale, IL, USA).

### 4.4. Anxiety Tests

#### 4.4.1. Novel Tank Diving Test (NTT)

The NTT was employed to evaluate both locomotor activity and anxiety-like behaviors in zebrafish, as described by Cachat et al. [35]. Individual fish were placed in a 1.5 L trapezoidal tank (15.2 × 27.9 × 7.1 cm), virtually divided into upper and lower zones. Behavior was recorded for 6 min and analyzed using ANY-maze^®^ software v. 7.48 (Stoelting Co., Wood Dale, IL, USA).

Locomotor activity was evaluated using multiple parameters, including time spent in the top zone (s), distance traveled within the top zone (m), total distance traveled (m), and average velocity (m/s). Anxiety-like behavior was assessed using established NTT metrics, such as the number of entries into the top zone, total time spent in the top zone (s), and latency to first entry into the top zone (s). These measures are widely recognized as reliable indicators of anxiety-related responses in zebrafish.

#### 4.4.2. Novel Approach Test (NAT)

The NAT was performed in a safe plastic arena with a diameter of 34 cm, a circumference of 108.5 cm, and a depth of 15 cm, following previously described protocols [36]. At the center of the arena, a multicolored LEGO^®^ figurine (2 × 4.25 cm) was placed to minimize potential biases due to color preference [37].

Individual zebrafish were observed for 5 min, and their behavior was recorded and analyzed using ANY-maze^®^ software v. 7.48 (Stoelting Co., Wood Dale, IL, USA). The arena was virtually divided into inner and outer zones using a centered circle of 17 cm diameter. Metrics recorded included: Time spent in each zone (s), Locomotor activity: distance traveled (m), and Immobility duration (s). These parameters provided quantitative measures of anxiety-like behavior and exploratory activity in zebrafish.

### 4.5. Biochemical and Enzymatic Evaluations

Following behavioral assessments, zebrafish were humanely euthanized in accordance with approved ethical protocols (e.g., overdose of tricaine methanesulfonate, MS-222) prior to brain tissue collection.

Brain tissues were homogenized at a 1:10 (*w*/*v*) ratio in ice-cold 0.1 M potassium phosphate buffer (pH 7.4) containing 1.15% KCl. Homogenization was performed for 1 min at 1000 rpm using a Mikro-Dismembrator U mill (Sartorius, New York, NY, USA) equipped with 3 mm magnetic balls (Sartorius Stedim Biotech GmbH, Goettingen, Germany). The homogenates were centrifuged at 14,000 rpm for 15 min, and the resulting supernatants were collected for biochemical analyses.

These analyses included the determination of total soluble protein content, enzymatic activities AChE, SOD, CAT, and GPX, as well as oxidative stress markers such as reduced GSH, MDA, and protein carbonyl levels. All procedures were performed according to established guidelines and previously reported protocols [38].

### 4.6. Molecular Docking

The Protein Data Bank provides 3D structures of the acetylcholine esterase enzyme (PDB ID: 4ey7) [39] and the human monoamine oxidase A enzyme (PDB ID: 2z5x) [28,39].

Molecular docking was carried out using Auto Dock Vina with an exhaustiveness value of 32 to ensure adequate sampling of ligand conformations [40], with both receptor and ligand files converted to pdbqt format. Prior to docking, the preparation of the two enzymes, their co-crystallized ligands, and the 29 bioactive compounds (α-Pinene, Sabinene, β-Pinene, Myrcene, δ-3-Carene, p-Cymene, Limonene, (E)-β-Ocimene, γ-Terpinene, Terpinolene, Linalool, cis-Limonene oxide, Citronellal, Terpinen-4-ol, α-Terpineol, Neral, Geraniol, Geranial, Neryl acetate, Geranyl acetate, (Z)-Caryophyllene, α-trans-Bergamotene, Germacrene D, (E, E)-α-Farnensene, δ-Cadinene, (E)-γ-Bisabolene, Caryophyllene oxide, (2E, 6Z)-Farnesol, and α-Sinensal) identified from *Citrus limon* EOs by Bouabdallah et al. [7] was performed using M.G.L. Tools [41]. The docking results were visualized and analyzed using Discovery Studio Visualizer 4.5 [42]. A 3D grid box measuring 50 × 50 × 50 Å (x, y, z) with a grid spacing of 0.375 Å was defined for both enzymes, centered at coordinates 40.58, 26.93, and −14.54 Å for human monoamine oxidase A and −11.14, −45.85, and 23.65 Å for acetylcholine esterase. Exhaustiveness was 32.

### 4.7. Statistical Analysis

All data are presented as mean ± standard error of the mean (SEM). Statistical analyses were performed using GraphPad Prism v9 software (GraphPad Software, San Diego, CA, USA). Comparisons among multiple groups were carried out using one-way analysis of variance (ANOVA) followed by Tukey’s post hoc test. For all analyses, a *p* value < 0.05 was considered statistically significant.

## 5. Conclusions

Collectively, the present findings demonstrate that *Citrus limon* leaf EOs exert significant neuroprotective effects in a zebrafish model of SCOP-induced cognitive impairment and anxiety-like behavior. Both HD (CEH) and SFME (CEM) oils effectively counteracted SCOP-induced deficits by improving behavioral performance, restoring cholinergic function, and reinforcing endogenous antioxidant defenses.

Importantly, despite sharing the same botanical origin, the extraction method emerged as a critical determinant of biological efficacy. The solvent-free microwave-extracted oil consistently exhibited superior antioxidant and cholinergic modulation, particularly at higher doses, highlighting how extraction-dependent compositional enrichment—especially in oxygenated monoterpenes—translates into enhanced *in vivo* neuroprotective outcomes.

By integrating behavioral, biochemical, and *in silico* molecular docking evidence, this study provides a coherent mechanistic framework linking cholinergic regulation and redox homeostasis to the observed cognitive and anxiolytic effects. Overall, *Citrus limon* leaf EOs, particularly when obtained through green extraction technologies, represent a promising candidate for further preclinical investigation in the context of neuroprotective research.

## Figures and Tables

**Table 1:** Types of interactions, amino acids involved, distance, and binding energy of the Co-crystal ligand (Donepezil), δ-Cadinene, and (*E*)-γ-Bisabolene within the Acetylcholinesterase enzyme active site.

Compound Name	Interaction/Amino Acid/Distance Å	Docking Energy Scores in kcal/mol
Co-crystal ligand (Donepezil)	H-Bond/Phe295/1.96 H-Bond/Ser293/3.06 Pi-Sigma/Trp286/3.64 Pi-Sigma/Tyr341/3.59 Pi-Pi Stacked/Trp86/4.46 Pi-Pi Stacked/Trp86/3.89 Pi-Pi Stacked/Trp286/5.11 Pi-Pi Stacked/Trp286/3.82 Pi-Pi Stacked/Tyr341/5.05 Pi-Alkyl/Tyr337/4.58 Pi-Alkyl/Phe338/5.05 Pi-Alkyl/Tyr341/4.90	−9.6
δ-Cadinene	Pi-Alkyl/Trp86/4.84 Pi-Alkyl/Trp86/4.98 Pi-Alkyl/Trp86/4.90 Pi-Alkyl/Trp86/4.21 Pi-Alkyl/Trp86/3.90 Pi-Alkyl/Tyr337/4.02 Pi-Alkyl/Tyr337/4.10 Pi-Alkyl/Phe338/5.15 Pi-Alkyl/Phe338/4.09 Pi-Alkyl/Tyr341/4.30 Pi-Alkyl/His447/5.03 Pi-Alkyl/His4474.67	−9.6
(*E*)-γ-Bisabolene	Pi-Sigma/Trp86/3.57 Pi-Sigma/Tyr337/3.49 Pi-Sigma/Phe338/3.59 Pi-Sigma/Tyr341/3.72 Alkyl/Val294/4.64 Pi-Alkyl/Trp86/5.17 Pi-Alkyl/Trp286/4.19 Pi-Alkyl/Tyr337/4.12 Pi-Alkyl/Phe338/5.27 Pi-Alkyl/Tyr341/4.21 Pi-Alkyl/Tyr341/4.78 Pi-Alkyl/Tyr341/4.80	−9.6

**Table 2:** Types of interactions, Amino acids involved, distance, and binding energy of Co-crystal ligand (Harmine), (E, E)-α-Farnensene, and (E)*-*γ-Bisabolene within the MAO A enzyme active site.

Compound Name	Interaction/Amino Acid/Distance Å	Docking Energy Scores in kcal/mol
Co-crystal ligand (Harmine)	Pi-Sigma/Tyr407/3.74 Pi-Sigma/Tyr444/3.65 Pi-Sigma/FAD600/3.66 Pi-Sigma/FAD600/3.72 Pi-Pi Stacked/Tyr407/4.29 Pi-Alkyl/Ile335/4.46 Pi-Alkyl/Leu337/5.40	−8.7
(E, E)-α-Farnensene	Pi-Sigma/Phe208/3.95 Alkyl/Leu97/4.98 Alkyl/Val210/4.71 Alkyl/Ile180/4.88 Alkyl/Ile335/4.66 Pi-Alkyl/Phe208/4.80 Pi-Alkyl/Tyr407/4.74 Pi-Alkyl/Tyr407/3.80 Pi-Alkyl/Tyr444/4.61 Pi-Alkyl/FAD600/4.13 Pi-Alkyl/FAD6004.27	−9.0
(E)-γ-Bisabolene	Pi-Sigma/Tyr407/3.95 Alkyl/Ile335/4.15 Alkyl/Leu337/4.37 Alkyl/Val210/4.94 Alkyl/Cys323/4.44 Alkyl/Ile335/5.34 Alkyl/Leu337/4.54 Alkyl/Cys323/4.71 Alkyl/Ile325/4.57 Alkyl/Ile335/5.42 Pi-Alkyl/Phe208/4.31 Pi-Alkyl/Phe352/5.40 Pi-Alkyl/Phe352/5.13 Pi-Alkyl/Tyr407/5.03	−8.9

## Data Availability

The original contributions presented in this study are included in the article. Further inquiries can be directed to the corresponding authors.

## Supplementary Materials

The following supporting information can be downloaded at https://www.mdpi.com/article/10.3390/ph19030458/s1. Table S1: Binding energy, interaction type, interacting amino acids, and bond distances obtained from molecular docking calculations of the tested essential oil compounds with the acetylcholinesterase (AChE) enzyme. Table S2: Binding energy, interaction type, interacting amino acids, and bond distances obtained from molecular docking calculations of the tested essential oil compounds with the monoamine oxidase A (MAO-A) enzyme.
