# Supplementary material for: Behavioral, Biochemical, and In Silico Evidence for Extraction-Dependent Neuroprotective Effects of Citrus limon Leaf Essential Oils in Scopolamine-Challenged Zebrafish
Source: Pharmaceuticals (Basel). 2026 Mar 11;19(3):458. doi: 10.3390/ph19030458 (PMC13029311; doi:10.3390/ph19030458)
Supplement: Supplementary file 1 [file pharmaceuticals-19-00458-s001.zip › pharmaceuticals-4129851-supplementary.pdf]

**Table S1:** Types of interactions, Amino acids involved, distance Å, and binding Energy of Co-crystal ligand and 29 compounds within the Acetylcholine esterase active site

| No. | Compound | Aminoacids | Type of bond | Distance (Å) | Scores Kcal/mol |
|---|---|---|---|---|---|
| | Donepezil | PHE295<br>SER293<br>TRP286<br>TYR341<br>TRP86<br>TRP86<br>TRP286<br>TRP286<br>TYR341<br>TYR337<br>PHE338<br>TYR341 | Conventional Hydrogen Bond<br>Carbon Hydrogen Bond<br>Pi-Sigma<br>Pi-Sigma<br>Pi-Pi Stacked<br>Pi-Pi Stacked<br>Pi-Pi Stacked<br>Pi-Pi Stacked<br>Pi-Pi Stacked<br>Pi-Alkyl<br>Pi-Alkyl<br>Pi-Alkyl | 1.96042<br>3.06831<br>3.64313<br>3.59437<br>4.46314<br>3.89021<br>5.11273<br>3.82202<br>5.05905<br>4.58978<br>5.05027<br>4.90695 | -9.6 |
| **1** | *α*-Pinene | A:TYR337<br>A:TRP86<br>A:TYR341<br>A:TRP86<br>A:TRP86<br>A:TRP86<br>A:TYR337<br>A:PHE338 | Pi-Sigma<br>Pi-Sigma<br>Pi-Sigma<br>Pi-Alkyl<br>Pi-Alkyl<br>Pi-Alkyl<br>Pi-Alkyl<br>Pi-Alkyl | 3.46724<br>3.60888<br>3.68171<br>4.07668<br>5.03225<br>4.12438<br>4.31153<br>4.64098 | -7.0 |
| **2** | Sabinene | A:TRP86<br>A:TRP86<br>A:TRP86<br>A:TRP86<br>A:TYR337<br>A:TYR337<br>A:PHE338<br>A:PHE338<br>A:TYR341<br>A:HIS447<br>A:HIS447 | Pi-Alkyl<br>Pi-Alkyl<br>Pi-Alkyl<br>Pi-Alkyl<br>Pi-Alkyl<br>Pi-Alkyl<br>Pi-Alkyl<br>Pi-Alkyl<br>Pi-Alkyl<br>Pi-Alkyl<br>Pi-Alkyl | 4.29864<br>3.91293<br>4.08488<br>4.12376<br>3.97238<br>4.51766<br>5.21954<br>5.01328<br>5.36066<br>5.07666<br>4.96961 | -7.2 |
| **3** | *β*-Pinene | A:TYR337<br>A:TYR337<br>A:PHE338<br>A:PHE338<br>A:PHE338<br>A:TYR341<br>A:TYR341<br>A:TYR341 | Pi-Alkyl<br>Pi-Alkyl<br>Pi-Alkyl<br>Pi-Alkyl<br>Pi-Alkyl<br>Pi-Alkyl<br>Pi-Alkyl<br>Pi-Alkyl | 4.36091<br>4.16724<br>4.87721<br>4.69428<br>4.75076<br>3.5387<br>5.18533<br>4.6781 | -6.8 |
| **4** | Myrcene | A:TRP286<br>A:TYR337<br>A:TYR337<br>A:PHE338<br>A:PHE338<br>A:TYR341<br>A:TYR341 | Pi-Alkyl<br>Pi-Alkyl<br>Pi-Alkyl<br>Pi-Alkyl<br>Pi-Alkyl<br>Pi-Alkyl<br>Pi-Alkyl | 4.37004<br>4.07122<br>3.6543<br>3.59887<br>4.18317<br>4.67423<br>4.19558 | -6.7 |

| | | | | | |
|---|---|---|---|---|---|
| | | A:HIS447 | Pi-Alkyl | 4.75682 | |
| **5** | δ-3-Carene | A:PHE338 | Pi-Sigma | 3.74951 | -7.1 |
| | | A:TRP86 | Pi-Sigma | 3.89117 | |
| | | A:TRP86 | Pi-Alkyl | 4.94447 | |
| | | A:TRP86 | Pi-Alkyl | 4.65936 | |
| | | A:TYR337 | Pi-Alkyl | 3.82459 | |
| | | A:HIS447 | Pi-Alkyl | 5.47898 | |
| | | A:HIS447 | Pi-Alkyl | 4.90426 | |
| **6** | *p*-Cymene | A:TRP86 | Pi-Sigma | 3.65347 | -7.3 |
| | | A:TRP86 | Pi-Sigma | 3.61294 | |
| | | A:PHE338 | Pi-Sigma | 3.72702 | |
| | | A:TYR337 | Pi-Pi Stacked | 3.71578 | |
| | | A:TRP86 | Pi-Pi T-shaped | 5.61871 | |
| | | A:TRP86 | Pi-Alkyl | 4.26492 | |
| | | A:TYR337 | Pi-Alkyl | 5.31501 | |
| | | A:TYR337 | Pi-Alkyl | 4.28358 | |
| | | A:TYR341 | Pi-Alkyl | 4.02604 | |
| | | A:HIS447 | Pi-Alkyl | 5.01059 | |
| **7** | Limonene | A:TYR337 | Pi-Sigma | 3.76328 | -7.1 |
| | | A:TYR124 | Pi-Alkyl | 5.20035 | |
| | | A:TRP286 | Pi-Alkyl | 4.81076 | |
| | | A:TYR337 | Pi-Alkyl | 5.19618 | |
| | | A:PHE338 | Pi-Alkyl | 4.10611 | |
| | | A:PHE338 | Pi-Alkyl | 4.92159 | |
| | | A:TYR341 | Pi-Alkyl | 4.92882 | |
| | | A:TYR341 | Pi-Alkyl | 4.17241 | |
| | | A:TYR341 | Pi-Alkyl | 4.17682 | |
| **8** | (*E*)-*β*-Ocimene | A:TRP86 | Pi-Sigma | 3.75753 | -6.9 |
| | | A:TYR337 | Pi-Sigma | 3.65845 | |
| | | A:TRP86 | Pi-Alkyl | 5.04992 | |
| | | A:TRP86 | Pi-Alkyl | 4.37582 | |
| | | A:TYR337 | Pi-Alkyl | 4.07936 | |
| | | A:PHE338 | Pi-Alkyl | 4.18686 | |
| | | A:PHE338 | Pi-Alkyl | 3.67763 | |
| | | A:TYR341 | Pi-Alkyl | 4.19631 | |
| | | A:HIS447 | Pi-Alkyl | 4.54605 | |
| **9** | *γ*-Terpinene | A:TRP86 | Pi-Sigma | 3.57386 | -7.1 |
| | | A:TRP86 | Pi-Sigma | 3.51853 | |
| | | A:PHE338 | Pi-Sigma | 3.69995 | |
| | | A:TYR337 | Pi-Pi Stacked | 3.80504 | |
| | | A:TRP86 | Pi-Alkyl | 4.11322 | |
| | | A:TYR337 | Pi-Alkyl | 4.18241 | |
| | | A:TYR341 | Pi-Alkyl | 4.05317 | |
| | | A:HIS447 | Pi-Alkyl | 5.32737 | |
| **10** | Terpinolene | A:TRP286 | Pi-Sigma | 3.70052 | -7.0 |
| | | A:TRP286 | Pi-Sigma | 3.99114 | |
| | | A:VAL294 | Alkyl | 4.92701 | |
| | | A:TRP286 | Pi-Alkyl | 5.04629 | |
| | | A:TRP286 | Pi-Alkyl | 3.91747 | |
| | | A:PHE338 | Pi-Alkyl | 5.05893 | |
| | | A:PHE338 | Pi-Alkyl | 5.26288 | |

| | | | | | |
|---|---|---|---|---|---|
| | | A:TYR341 | Pi-Alkyl | 4.585 | |
| | | A:TYR341 | Pi-Alkyl | 4.94859 | |
| | | A:TYR341 | Pi-Alkyl | 4.19427 | |
| **11** | Linalool | A:TRP86 | Pi-Donor Hydrogen Bond | 4.18484 | -6.3 |
| | | A:TYR337 | Pi-Sigma | 3.66402 | |
| | | A:TYR337 | Pi-Alkyl | 4.22265 | |
| | | A:PHE338 | Pi-Alkyl | 4.10431 | |
| | | A:TYR341 | Pi-Alkyl | 4.2566 | |
| **12** | *cis*-Limonene oxide | A:TYR337 | Pi-Sigma | 3.7629 | -7.2 |
| | | A:TYR337 | Pi-Alkyl | 5.30054 | |
| | | A:PHE338 | Pi-Alkyl | 4.93485 | |
| | | A:PHE338 | Pi-Alkyl | 4.13547 | |
| | | A:PHE338 | Pi-Alkyl | 4.66355 | |
| | | A:TYR341 | Pi-Alkyl | 4.32498 | |
| | | A:TYR341 | Pi-Alkyl | 4.7713 | |
| | | A:HIS447 | Pi-Alkyl | 4.83994 | |
| **13** | Citronellal | A:TRP86 | Pi-Sigma | 3.58194 | -6.4 |
| | | A:TYR337 | Pi-Sigma | 3.62172 | |
| | | A:TYR337 | Pi-Alkyl | 4.3033 | |
| | | A:PHE338 | Pi-Alkyl | 4.19643 | |
| | | A:PHE338 | Pi-Alkyl | 4.17797 | |
| | | A:TYR341 | Pi-Alkyl | 4.26526 | |
| | | A:HIS447 | Pi-Alkyl | 4.69918 | |
| **14** | Terpinen-4-ol | A:PHE338 | Pi-Sigma | 3.69443 | -7.3 |
| | | A:TRP86 | Pi-Sigma | 3.55259 | |
| | | A:TYR337 | Pi-Alkyl | 3.64786 | |
| | | A:PHE338 | Pi-Alkyl | 4.51437 | |
| | | A:TYR341 | Pi-Alkyl | 5.26485 | |
| **15** | $\alpha$-Terpineol | A:TRP286 | Pi-Alkyl | 5.00044 | -7.1 |
| | | A:TYR341 | Pi-Alkyl | 4.41219 | |
| **16** | Neral | A:ARG296:HN | Conventional Hydrogen Bond | 2.31759 | -6.7 |
| | | A:TYR337 | Pi-Sigma | 3.82893 | |
| | | A:PHE338 | Pi-Sigma | 3.57295 | |
| | | A:TYR337 | Pi-Sigma | 3.69959 | |
| | | A:TYR124 | Pi-Alkyl | 5.13507 | |
| | | A:TRP286 | Pi-Alkyl | 4.43107 | |
| | | A:PHE338 | Pi-Alkyl | 4.56683 | |
| | | A:TYR341 | Pi-Alkyl | 4.28767 | |
| **17** | Geraniol | A:GLY121:HN | Conventional Hydrogen Bond | 2.77634 | -6.9 |
| | | A:SER203:HG | Conventional Hydrogen Bond | 2.14138 | |
| | | A:GLU202:OE1 | Conventional Hydrogen Bond | 3.21889 | |
| | | A:TYR337 | Pi-Sigma | 3.62004 | |
| | | A:TRP86 | Pi-Sigma | 3.9984 | |
| | | A:TRP86 | Pi-Sigma | 3.5784 | |
| | | A:TYR337 | Pi-Alkyl | 4.53374 | |
| | | A:PHE338 | Pi-Alkyl | 4.10685 | |
| | | A:TYR341 | Pi-Alkyl | 4.13363 | |
| | | A:HIS447 | Pi-Alkyl | 5.39054 | |
| **18** | Geranial (citral) | A:TYR133:HH | Conventional Hydrogen Bond | 2.59923 | -6.9 |
| | | A:TYR337 | Pi-Sigma | 3.6676 | |
| | | A:TRP86 | Pi-Sigma | 3.56769 | |

| | | | | | |
|---|---|---|---|---|---|
| | | A:TRP86<br>A:TYR337<br>A:PHE338<br>A:TYR341<br>A:HIS447 | Pi-Alkyl<br>Pi-Alkyl<br>Pi-Alkyl<br>Pi-Alkyl<br>Pi-Alkyl | 4.17415<br>4.37194<br>4.15884<br>4.28471<br>5.23829 | |
| **19** | Neryl acetate | A:GLY121:HN<br>A:GLY122:HN<br>A:SER203:HG<br>A:HIS447:HE2<br>A:TYR337<br>A:TRP86<br>A:TRP86<br>A:TYR337<br>A:PHE338<br>A:TYR341<br>A:HIS447 | Conventional Hydrogen Bond<br>Conventional Hydrogen Bond<br>Conventional Hydrogen Bond<br>Conventional Hydrogen Bond<br>Pi-Sigma<br>Pi-Sigma<br>Pi-Alkyl<br>Pi-Alkyl<br>Pi-Alkyl<br>Pi-Alkyl<br>Pi-Alkyl | 2.62878<br>2.56401<br>2.20499<br>2.5538<br>3.6565<br>3.59225<br>4.59634<br>4.42442<br>4.65312<br>4.27522<br>4.97051 | -7.3 |
| **20** | Geranyl acetate | A:TYR124:HH<br>A:TRP286<br>A:PHE338<br>A:TRP86<br>A:TRP286<br>A:TYR337<br>A:TYR341<br>A:TYR341 | Conventional Hydrogen Bond<br>Pi-Sigma<br>Pi-Sigma<br>Pi-Sigma<br>Pi-Alkyl<br>Pi-Alkyl<br>Pi-Alkyl<br>Pi-Alkyl | 2.91531<br>3.91708<br>3.91643<br>3.76353<br>4.92833<br>4.23046<br>5.04835<br>4.29407 | -7.0 |
| **21** | (Z)-Caryophyllene | A:TRP86<br>A:TRP86<br>A:TRP86<br>A:TRP86<br>A:TYR337<br>A:TYR337<br>A:TYR337<br>A:PHE338<br>A:PHE338<br>A:TYR341<br>A:TYR341 | Pi-Alkyl<br>Pi-Alkyl<br>Pi-Alkyl<br>Pi-Alkyl<br>Pi-Alkyl<br>Pi-Alkyl<br>Pi-Alkyl<br>Pi-Alkyl<br>Pi-Alkyl<br>Pi-Alkyl<br>Pi-Alkyl | 5.06745<br>3.52199<br>5.17444<br>3.85378<br>4.22846<br>3.87628<br>4.08391<br>4.57649<br>4.09379<br>4.54944<br>4.32568 | -8.1 |
| **22** | *α-trans*-Bergamotene | A:TYR337<br>A:TRP86<br>A:TRP86<br>A:TRP86<br>A:TYR124<br>A:TYR124<br>A:TRP286<br>A:PHE297<br>A:PHE297<br>A:TYR337<br>A:PHE338<br>A:PHE338<br>A:TYR341<br>A:HIS447 | Pi-Sigma<br>Pi-Alkyl<br>Pi-Alkyl<br>Pi-Alkyl<br>Pi-Alkyl<br>Pi-Alkyl<br>Pi-Alkyl<br>Pi-Alkyl<br>Pi-Alkyl<br>Pi-Alkyl<br>Pi-Alkyl<br>Pi-Alkyl<br>Pi-Alkyl<br>Pi-Alkyl | 3.62165<br>4.79024<br>4.42386<br>5.15308<br>5.08954<br>5.19914<br>4.8299<br>5.48264<br>4.68938<br>4.88539<br>4.10335<br>4.57001<br>4.76614<br>4.84763 | -8.5 |
| **23** | Germacrene D | A:TRP86<br>A:TRP86 | Pi-Sigma<br>Pi-Alkyl | 3.93048<br>4.47066 | -8.8 |

| | | | | | |
|---|---|---|---|---|---|
| | | A:PHE295<br>A:PHE297<br>A:TYR337<br>A:PHE338<br>A:HIS447<br>A:HIS447 | Pi-Alkyl<br>Pi-Alkyl<br>Pi-Alkyl<br>Pi-Alkyl<br>Pi-Alkyl<br>Pi-Alkyl | 5.03936<br>4.4874<br>5.49218<br>4.86552<br>5.15677<br>4.93789 | |
| **24** | (*E*, *E*)-α-Farnensene | A:VAL294<br>A:TRP86<br>A:TRP86<br>A:TRP86<br>A:TYR337<br>A:PHE338<br>A:TYR341<br>A:TYR341 | Alkyl<br>Pi-Alkyl<br>Pi-Alkyl<br>Pi-Alkyl<br>Pi-Alkyl<br>Pi-Alkyl<br>Pi-Alkyl<br>Pi-Alkyl | 4.62616<br>4.26466<br>4.36611<br>4.40377<br>4.62813<br>5.35397<br>4.63552<br>4.88907 | -8.3 |
| **25** | *δ*-Cadinene | A:TRP86<br>A:TRP86<br>A:TRP86<br>A:TRP86<br>A:TRP86<br>A:TYR337<br>A:TYR337<br>A:PHE338<br>A:PHE338<br>A:TYR341<br>A:HIS447<br>A:HIS447 | Pi-Alkyl<br>Pi-Alkyl<br>Pi-Alkyl<br>Pi-Alkyl<br>Pi-Alkyl<br>Pi-Alkyl<br>Pi-Alkyl<br>Pi-Alkyl<br>Pi-Alkyl<br>Pi-Alkyl<br>Pi-Alkyl<br>Pi-Alkyl | 4.84698<br>4.98202<br>4.90915<br>4.21764<br>3.90465<br>4.02154<br>4.10196<br>5.1591<br>4.09958<br>4.30482<br>5.03791<br>4.67224 | -9.6 |
| **26** | (*E*)-*γ*-Bisabolene | A:TRP86<br>A:TYR337<br>A:PHE338<br>A:TYR341<br>A:VAL294<br>A:TRP86<br>A:TRP286<br>A:TYR337<br>A:PHE338<br>A:TYR341<br>A:TYR341<br>A:TYR341 | Pi-Sigma<br>Pi-Sigma<br>Pi-Sigma<br>Pi-Sigma<br>Alkyl<br>Pi-Alkyl<br>Pi-Alkyl<br>Pi-Alkyl<br>Pi-Alkyl<br>Pi-Alkyl<br>Pi-Alkyl<br>Pi-Alkyl | 3.57731<br>3.49462<br>3.59422<br>3.7213<br>4.64116<br>5.17064<br>4.19827<br>4.1251<br>5.27963<br>4.21052<br>4.78465<br>4.80103 | -9.6 |
| **27** | Caryophyllene oxide | A:TRP86<br>A:TRP86<br>A:TRP86<br>A:PHE297<br>A:TYR337<br>A:PHE338<br>A:HIS447<br>A:HIS447 | Pi-Alkyl<br>Pi-Alkyl<br>Pi-Alkyl<br>Pi-Alkyl<br>Pi-Alkyl<br>Pi-Alkyl<br>Pi-Alkyl<br>Pi-Alkyl | 4.88526<br>4.50141<br>4.26291<br>4.52158<br>5.15375<br>5.00145<br>4.78844<br>4.314 | -8.5 |
| **28** | (2*E,*6*Z*) -Farnesol | A:TYR124:OH<br>A:TRP86<br>A:TRP86<br>A:PHE295<br>A:PHE297 | Conventional Hydrogen Bond<br>Pi-Sigma<br>Pi-Alkyl<br>Pi-Alkyl<br>Pi-Alkyl | 2.93466<br>3.70228<br>3.76552<br>4.57333<br>4.90514 | -7.6 |

| | | | | | |
|---|---|---|---|---|---|
| | | A:TYR337<br>A:PHE338<br>A:TYR341<br>A:HIS447 | Pi-Alkyl<br>Pi-Alkyl<br>Pi-Alkyl<br>Pi-Alkyl | 4.69641<br>4.87844<br>4.10154<br>4.4583 | |
| **29** | α-Sinensal | A:TYR133:HH<br>A:TYR337<br>A:TRP86<br>A:TYR124<br>A:TRP286<br>A:PHE297<br>A:PHE338<br>A:TYR341 | Conventional Hydrogen Bond<br>Pi-Sigma<br>Pi-Sigma<br>Pi-Alkyl<br>Pi-Alkyl<br>Pi-Alkyl<br>Pi-Alkyl<br>Pi-Alkyl | 2.8444<br>3.84971<br>3.88064<br>5.30369<br>4.94247<br>4.89652<br>4.48565<br>4.83767 | -8.5 |

**Table S2:** Types of interactions, Amino acids involved, distance Å , and binding energy of Co-crystal ligand and 29 compounds within MAO A enzyme active site

| No. | Compound | Aminoacids | Type of bond | Distance (Å) | Scores Kcal/mol |
|---|---|---|---|---|---|
| | Co-crystal ligand (Harmine) | A:TYR407<br>A:TYR444<br>A:FAD600<br>A:FAD600<br>A:TYR407<br>A:ILE335<br>A:LEU337 | Pi-Sigma<br>Pi-Sigma<br>Pi-Sigma<br>Pi-Sigma<br>Pi-Pi Stacked<br>Pi-Alkyl<br>Pi-Alkyl | 3.74741<br>3.65891<br>3.66149<br>3.7215<br>4.29587<br>4.46164<br>5.40743 | -8.7 |
| **1** | *α*-Pinene | A:ILE180<br>A:FAD600<br>A:PHE208<br>A:PHE352<br>A:PHE352<br>A:TYR69<br>A:PHE208<br>A:ILE180<br>A:ILE335<br>A:ILE180<br>A:ILE335 | Alkyl<br>Pi-Alkyl<br>Pi-Alkyl<br>Pi-Alkyl<br>Pi-Alkyl<br>Pi-Alkyl<br>Pi-Alkyl<br>Alkyl<br>Alkyl<br>Alkyl<br>Alkyl | 5.47983<br>5.45961<br>5.28305<br>5.17401<br>4.93397<br>4.89726<br>4.70705<br>4.70685<br>4.55398<br>4.52388<br>4.29595 | -6.8 |
| **2** | Sabinene | A:ILE180<br>A:ILE335<br>A:ILE180<br>A:ILE335<br>A:ILE335<br>A:LEU337<br>A:PHE208<br>A:PHE352 | Alkyl<br>Alkyl<br>Alkyl<br>Alkyl<br>Alkyl<br>Alkyl<br>Pi-Alkyl<br>Pi-Alkyl | 4.95368<br>4.10465<br>4.6954<br>4.77951<br>4.66662<br>4.42918<br>4.18856<br>4.64678 | -6.6 |
| **3** | *β*-Pinene | A:ILE335<br>A:LEU337<br>A:VAL210<br>A:LEU337<br>A:ILE180<br>A:ILE335 | Alkyl<br>Alkyl<br>Alkyl<br>Alkyl<br>Alkyl<br>Alkyl | 4.01477<br>5.37843<br>5.26809<br>4.05732<br>4.04202<br>3.44819 | -5.4 |

| | | | | | |
|---|---|---|---|---|---|
| | | A:TYR69 | Pi-Alkyl | 4.48746 | |
| | | A:PHE352 | Pi-Alkyl | 5.06061 | |
| | | A:PHE352 | Pi-Alkyl | 5.20965 | |
| | | A:TYR407 | Pi-Alkyl | 5.28462 | |
| **4** | Myrcene | A:VAL210 | Alkyl | 5.2876 | -6.4 |
| | | A:CYS323 | Alkyl | 4.83501 | |
| | | A:ILE335 | Alkyl | 4.95778 | |
| | | A:LEU337 | Alkyl | 4.17932 | |
| | | A:TYR407 | Pi-Alkyl | 5.09023 | |
| | | A:TYR407 | Pi-Alkyl | 3.8806 | |
| | | A:TYR444 | Pi-Alkyl | 5.43117 | |
| | | A:FAD600 | Pi-Alkyl | 4.33583 | |
| | | A:FAD600 | Pi-Alkyl | 4.34771 | |
| **5** | δ-3-Carene | A:ILE335 | Alkyl | 5.2869 | -5.5 |
| | | A:LEU337 | Alkyl | 4.33265 | |
| | | A:ILE335 | Alkyl | 3.27833 | |
| | | A:LEU337 | Alkyl | 3.89048 | |
| | | A:MET350 | Alkyl | 5.33071 | |
| | | A:PHE352 | Pi-Alkyl | 4.83939 | |
| **6** | *p*-Cymene | A:TYR407 | Pi-Sigma | 3.83399 | -7.3 |
| | | A:TYR407 | Pi-Pi Stacked | 4.99717 | |
| | | A:ILE180 | Alkyl | 5.0785 | |
| | | A:ILE335 | Alkyl | 4.4767 | |
| | | A:TYR444 | Pi-Alkyl | 4.71223 | |
| | | A:FAD600 | Pi-Alkyl | 4.0502 | |
| | | A:FAD600 | Pi-Alkyl | 4.19541 | |
| **7** | Limonene | A:TYR407 | Pi-Sigma | 3.66396 | -7.2 |
| | | A:ILE335 | Alkyl | 3.83647 | |
| | | A:LEU337 | Alkyl | 4.66527 | |
| | | A:MET350 | Alkyl | 5.44631 | |
| | | A:ILE180 | Alkyl | 4.73552 | |
| | | A:ILE335 | Alkyl | 3.969 | |
| | | A:PHE352 | Pi-Alkyl | 5.31431 | |
| | | A:PHE352 | Pi-Alkyl | 4.76972 | |
| | | A:TYR407 | Pi-Alkyl | 5.01014 | |
| **8** | (*E*)-*β*-Ocimene | A:VAL210 | Alkyl | 4.92116 | -6.6 |
| | | A:CYS323 | Alkyl | 4.89839 | |
| | | A:LEU337 | Alkyl | 4.37223 | |
| | | A:ILE180 | Alkyl | 5.46342 | |
| | | A:PHE352 | Pi-Alkyl | 5.45578 | |
| | | A:TYR407 | Pi-Alkyl | 5.27993 | |
| | | A:TYR407 | Pi-Alkyl | 4.07761 | |
| | | A:FAD600 | Pi-Alkyl | 4.61393 | |
| | | A:FAD600 | Pi-Alkyl | 4.55458 | |
| **9** | *γ*-Terpinene | A:TYR407 | Pi-Sigma | 3.84575 | -7.3 |
| | | A:TYR407 | Pi-Pi Stacked | 4.93992 | |
| | | A:ILE180 | Alkyl | 5.11248 | |
| | | A:ILE335 | Alkyl | 4.51148 | |
| | | A:TYR444 | Pi-Alkyl | 4.57165 | |
| | | A:FAD600 | Pi-Alkyl | 4.01272 | |
| | | A:FAD600 | Pi-Alkyl | 4.18678 | |

| | | | | | |
|---|---|---|---|---|---|
| **10** | Terpinolene | A:PHE352<br>A:TYR407<br>A:TYR407<br>A:TYR444<br>A:TYR444<br>A:FAD600<br>A:FAD600 | Pi-Alkyl<br>Pi-Alkyl<br>Pi-Alkyl<br>Pi-Alkyl<br>Pi-Alkyl<br>Pi-Alkyl<br>Pi-Alkyl | 5.13009<br>4.28288<br>4.16015<br>5.18844<br>4.73984<br>4.13147<br>4.42424 | -7.3 |
| **11** | Linalool | A:GLN215:HE21<br>A:PHE208:O<br>A:TYR69<br>A:PHE352 | Conventional Hydrogen Bond<br>Conventional Hydrogen Bond<br>Pi-Alkyl<br>Pi-Alkyl | 2.20342<br>3.09594<br>4.81654<br>4.8728 | -6.3 |
| **12** | *cis*-Limonene oxide | A:ILE335<br>A:ILE335<br>A:LEU337<br>A:PHE352<br>A:PHE352<br>A:TYR407<br>A:FAD600<br>A:FAD600 | Alkyl<br>Alkyl<br>Alkyl<br>Pi-Alkyl<br>Pi-Alkyl<br>Pi-Alkyl<br>Pi-Alkyl<br>Pi-Alkyl | 5.163<br>3.86101<br>3.92574<br>5.37136<br>5.32345<br>3.94817<br>4.63991<br>4.51194 | -6.9 |
| **13** | Citronellal | A:ILE180<br>A:ILE335<br>A:VAL210<br>A:CYS323<br>A:ILE335<br>A:LEU337<br>Pi-Alkyl | Alkyl<br>Alkyl<br>Alkyl<br>Alkyl<br>Alkyl<br>Alkyl<br>Pi-Alkyl | 4.9741<br>4.48937<br>5.15741<br>4.70268<br>5.08048<br>4.29054<br>4.81628 | -6.3 |
| **14** | Terpinen-4-ol | A:TYR407:HH<br>A:TYR407<br>A:TYR407 | Conventional Hydrogen Bond<br>Pi-Sigma<br>Pi-Alkyl | 2.35295<br>3.86997<br>4.69013 | -6.8 |
| **15** | *α*-Terpineol | A:TYR407<br>A:FAD600<br>A:FAD600<br>A:TYR407 | Pi-Sigma<br>Pi-Sigma<br>Pi-Sigma<br>Pi-Alkyl | 3.7563<br>3.82034<br>3.99546<br>4.47582 | -7.0 |
| **16** | Neral | A:ILE335<br>A:LEU337<br>A:ILE180 | Alkyl<br>Alkyl<br>Alkyl | 4.73297<br>4.0085<br>5.37232 | -6.5 |
| **17** | Geraniol | A:FAD600:H5<br>A:TYR407<br>A:FAD600<br>A:FAD600<br>A:VAL210<br>A:CYS323<br>A:ILE335<br>A:LEU337<br>A:TYR407 | Conventional Hydrogen Bond<br>Pi-Donor Hydrogen Bond<br>Pi-Donor Hydrogen Bond<br>Pi-Donor Hydrogen Bond<br>Alkyl<br>Alkyl<br>Alkyl<br>Alkyl<br>Pi-Alkyl | 2.89681<br>3.36931<br>4.01829<br>3.71142<br>5.0132<br>4.81095<br>5.25385<br>4.3604<br>5.27631 | -6.7 |
| **18** | Geranial (citral) | A:VAL210<br>A:CYS323<br>A:ILE335<br>A:LEU337<br>A:TYR407 | Alkyl<br>Alkyl<br>Alkyl<br>Alkyl<br>Pi-Alkyl | 5.07046<br>4.7714<br>5.18728<br>4.34682<br>5.26657 | -6.8 |
| **19** | Neryl acetate | A:TYR444:HH | Conventional Hydrogen Bond | 2.35937 | -7.1 |

| | | | | | |
|---|---|---|---|---|---|
| | | A:TYR407 | Pi-Sigma | 3.97231 | |
| | | A:CYS323 | Alkyl | 4.7018 | |
| | | A:ILE325 | Alkyl | 4.59875 | |
| | | A:ILE335 | Alkyl | 5.42978 | |
| | | A:ILE335 | Alkyl | 3.97885 | |
| | | A:LEU337 | Alkyl | 4.23612 | |
| | | A:PHE208 | Pi-Alkyl | 4.33929 | |
| **20** | Geranyl acetate | A:TYR444:HH | Conventional Hydrogen Bond | 2.60114 | -7.2 |
| | | A:TYR407 | Pi-Sigma | 3.66859 | |
| | | A:TYR444 | Pi-Sigma | 3.83872 | |
| | | A:FAD600 | Pi-Sigma | 3.7139 | |
| | | A:FAD600 | Pi-Sigma | 3.8078 | |
| | | A:VAL210 | Alkyl | 5.17031 | |
| | | A:CYS323 | Alkyl | 4.65323 | |
| | | A:ILE335 | Alkyl | 5.23874 | |
| | | A:LEU337 | Alkyl | 4.89588 | |
| | | A:ILE180 | Alkyl | 5.49231 | |
| | | A:PHE352 | Pi-Alkyl | 5.20214 | |
| | | A:TYR407 | Pi-Alkyl | 5.39909 | |
| **21** | (Z)-Caryophyllene | A:ILE180 | Alkyl | 5.04857 | -5.4 |
| | | A:ILE335 | Alkyl | 3.78361 | |
| | | A:LEU337 | Alkyl | 4.2249 | |
| | | A:ILE335 | Alkyl | 3.35723 | |
| | | A:LEU337 | Alkyl | 4.82173 | |
| | | A:MET350 | Alkyl | 5.00374 | |
| | | A:PHE352 | Pi-Alkyl | 5.39405 | |
| | | A:PHE352 | Pi-Alkyl | 5.14596 | |
| | | A:PHE352 | Pi-Alkyl | 4.30897 | |
| | | A:TYR407 | Pi-Alkyl | 4.72098 | |
| **22** | *α-trans*-Bergamotene | A:ILE335 | Alkyl | 4.58033 | -6.1 |
| | | A:LEU337 | Alkyl | 4.42505 | |
| | | A:LEU97 | Alkyl | 5.36243 | |
| | | A:CYS323 | Alkyl | 4.65401 | |
| | | A:ILE325 | Alkyl | 4.24871 | |
| | | A:VAL210 | Alkyl | 4.56338 | |
| | | A:CYS323 | Alkyl | 4.45677 | |
| | | A:LEU337 | Alkyl | 5.24373 | |
| | | A:PHE208 | Pi-Alkyl | 4.44993 | |
| | | A:PHE352 | Pi-Alkyl | 4.51296 | |
| **23** | Germacrene D | A:TYR407 | Pi-Sigma | 3.73036 | -6.8 |
| | | A:ILE335 | Alkyl | 5.11113 | |
| | | A:ILE180 | Alkyl | 4.8854 | |
| | | A:PHE208 | Pi-Alkyl | 3.8869 | |
| | | A:TYR407 | Pi-Alkyl | 4.12265 | |
| | | A:TYR444 | Pi-Alkyl | 5.12992 | |
| | | A:TYR444 | Pi-Alkyl | 5.24789 | |
| | | A:FAD600 | Pi-Alkyl | 4.15632 | |
| | | A:FAD600 | Pi-Alkyl | 4.16868 | |
| **24** | (*E*, *E*)-α-Farnensene | A:PHE208 | Pi-Sigma | 3.95507 | -8.9 |
| | | A:LEU97 | Alkyl | 4.9815 | |
| | | A:VAL210 | Alkyl | 4.71932 | |

| | | | | | |
|---|---|---|---|---|---|
| | | A:ILE180<br>A:ILE335<br>A:PHE208<br>A:TYR407<br>A:TYR407<br>A:TYR444<br>A:FAD600<br>A:FAD600 | Alkyl<br>Alkyl<br>Pi-Alkyl<br>Pi-Alkyl<br>Pi-Alkyl<br>Pi-Alkyl<br>Pi-Alkyl<br>Pi-Alkyl | 4.8841<br>4.66254<br>4.80176<br>4.74977<br>3.80653<br>4.61922<br>4.13234<br>4.27979 | |
| **25** | *δ*-Cadinene | A:TYR407<br>A:ILE180<br>A:ILE335<br>A:ILE180<br>A:TYR69<br>A:PHE208<br>A:PHE352<br>A:TYR444<br>A:FAD600<br>A:FAD600<br>A:FAD600<br>A:FAD600 | Pi-Sigma<br>Alkyl<br>Alkyl<br>Alkyl<br>Pi-Alkyl<br>Pi-Alkyl<br>Pi-Alkyl<br>Pi-Alkyl<br>Pi-Alkyl<br>Pi-Alkyl<br>Pi-Alkyl<br>Pi-Alkyl | 3.58775<br>5.48021<br>4.59699<br>4.60504<br>4.41385<br>4.11983<br>5.03975<br>5.2676<br>4.1119<br>5.09677<br>4.01942<br>5.18685 | -6.9 |
| **26** | (*E*)-*γ*-Bisabolene | A:TYR407<br>A:ILE335<br>A:LEU337<br>A:VAL210<br>A:CYS323<br>A:ILE335<br>A:LEU337<br>A:CYS323<br>A:ILE325<br>A:ILE335<br>A:PHE208<br>A:PHE352<br>A:PHE352<br>A:TYR407 | Pi-Sigma<br>Alkyl<br>Alkyl<br>Alkyl<br>Alkyl<br>Alkyl<br>Alkyl<br>Alkyl<br>Alkyl<br>Alkyl<br>Pi-Alkyl<br>Pi-Alkyl<br>Pi-Alkyl<br>Pi-Alkyl | 3.95359<br>4.15216<br>4.37573<br>4.94074<br>4.44778<br>5.34889<br>4.54304<br>4.71998<br>4.57251<br>5.4286<br>4.31805<br>5.40546<br>5.13703<br>5.03991 | -9.0 |
| **27** | Caryophyllene oxide | A:TYR407:HH<br>A:ASN181:CA<br>A:ILE180<br>A:ILE335<br>A:LEU337<br>A:ILE335<br>A:LEU337<br>A:MET350<br>A:LEU337<br>A:MET350<br>A:ILE335<br>A:TYR69<br>A:PHE208<br>A:PHE352 | Conventional Hydrogen Bond<br>Carbon Hydrogen Bond<br>Alkyl<br>Alkyl<br>Alkyl<br>Alkyl<br>Alkyl<br>Alkyl<br>Alkyl<br>Alkyl<br>Alkyl<br>Pi-Alkyl<br>Pi-Alkyl<br>Pi-Alkyl | 3.05117<br>3.75097<br>5.02368<br>4.17779<br>4.39154<br>3.63697<br>4.75909<br>4.91519<br>3.93689<br>5.27699<br>5.14852<br>3.95888<br>3.76203<br>4.23365 | -6.7 |
| **28** | (2*E*,6*Z*) -Farnesol | A:VAL210:HN<br>A:ALA111:CA<br>A:ALA111:O | Conventional Hydrogen Bond<br>Carbon Hydrogen Bond<br>Carbon Hydrogen Bond | 2.22668<br>3.63161<br>3.43175 | -7.4 |

| | | | | | |
|---|---|---|---|---|---|
| | | A:PHE208<br>A:ILE335<br>A:LEU337<br>A:MET350<br>A:CYS323<br>A:ILE325<br>A:ILE335<br>A:TYR69<br>A:PHE352<br>A:TYR407<br>A:TYR444 | Pi-Sigma<br>Alkyl<br>Alkyl<br>Alkyl<br>Alkyl<br>Alkyl<br>Alkyl<br>Pi-Alkyl<br>Pi-Alkyl<br>Pi-Alkyl<br>Pi-Alkyl | 3.82452<br>4.77015<br>4.72008<br>5.06563<br>5.21709<br>4.62719<br>5.42173<br>5.10053<br>4.39959<br>4.34645<br>4.61841 | |
| **29** | α-Sinensal | A:VAL210:HN<br>A:ALA111:O<br>A:PHE208<br>A:ILE180<br>A:PHE352<br>A:TYR407<br>A:FAD600<br>A:FAD600 | Conventional Hydrogen Bond<br>Carbon Hydrogen Bond<br>Pi-Sigma<br>Alkyl<br>Pi-Alkyl<br>Pi-Alkyl<br>Pi-Alkyl<br>Pi-Alkyl | 2.4674<br>3.41221<br>3.95206<br>5.38016<br>5.33654<br>3.96783<br>4.54235<br>4.40371 | -7.7 |
